# Supplementary material for: Predicting gene expression using morphological cell responses to nanotopography
Source: Nat Commun. 2020 Mar 13;11:1384. doi: 10.1038/s41467-020-15114-1 (PMC7070086; doi:10.1038/s41467-020-15114-1)
Supplement: Supplementary file 1 — Supplementary Information [file 41467_2020_15114_MOESM1_ESM.pdf]

Supplementary Information

Predicting gene expression using morphological cell responses to nanotopography

Cutiongco et al.

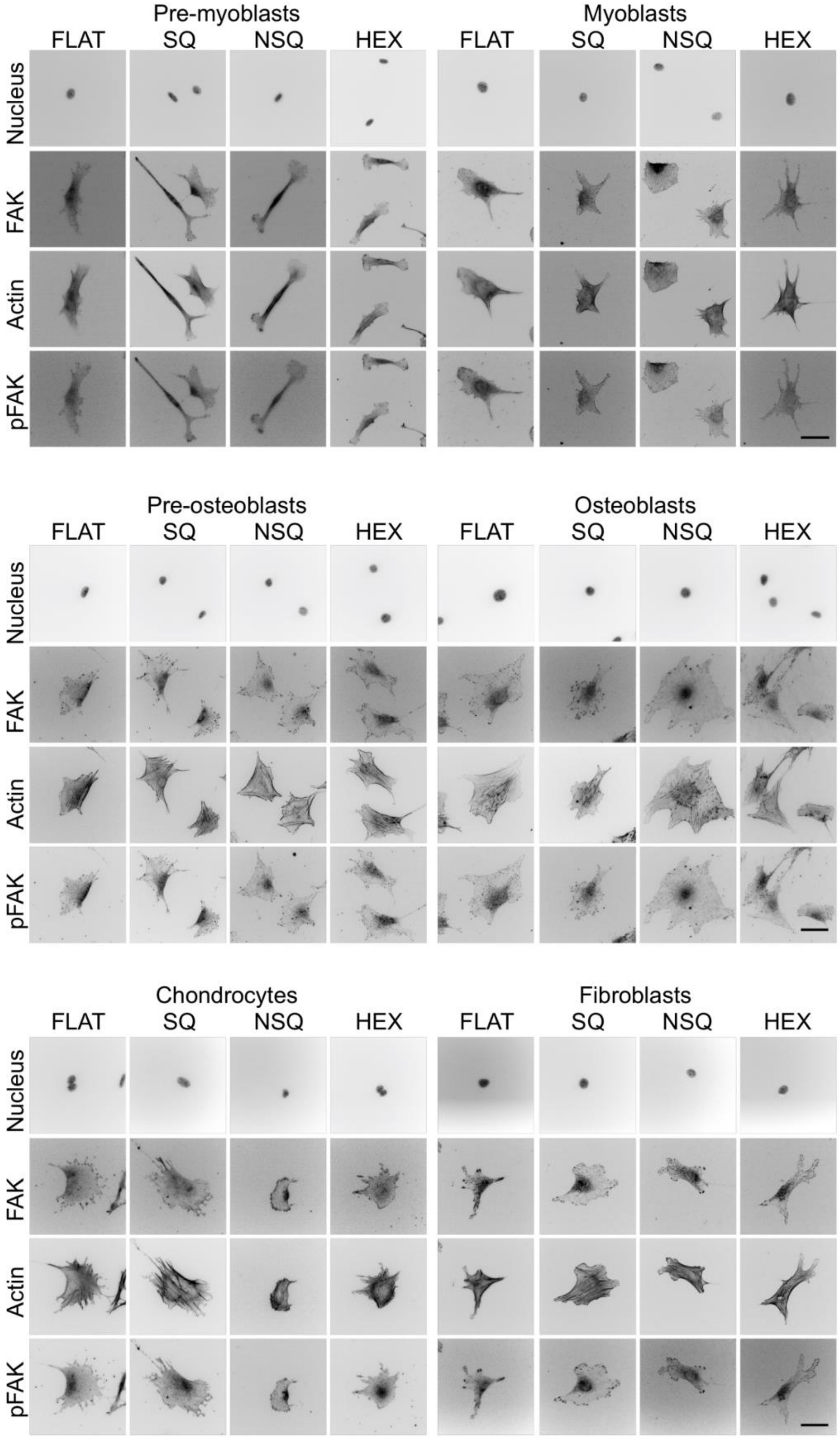

Supplementary Figure 1. Representative images of musculoskeletal cell types cultured on four different nanotopographies. Cells were stained using antibodies against the nucleus, actin cytoskeleton, focal adhesion kinase (FAK) and phosphorylated (Y397) focal adhesion kinase (pFAK) and imaged after 2 days in culture on the nanotopographies: FLAT, SQ, NSQ, and HEX. Scale bar = 50 µm.

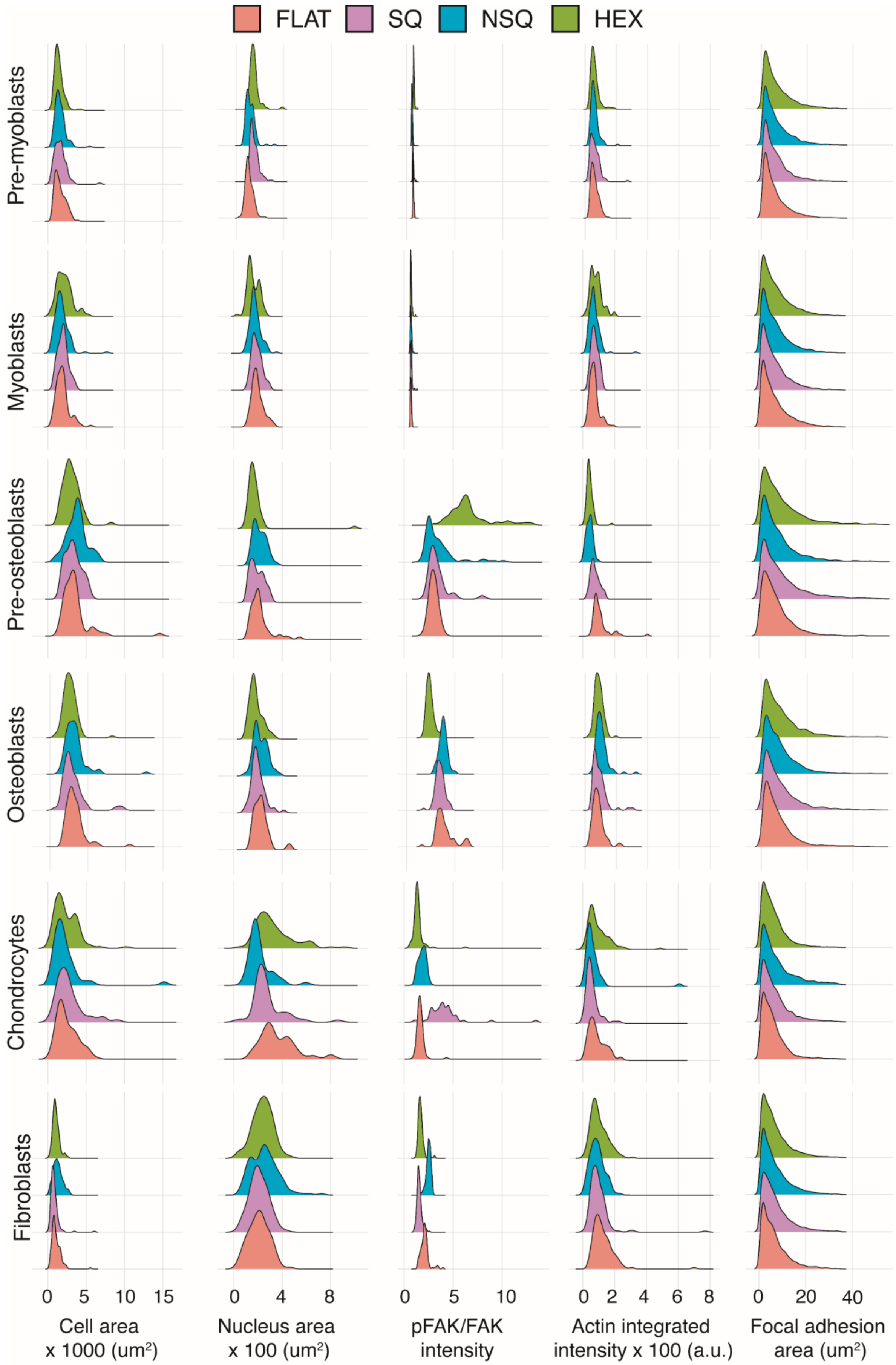
FLAT
SQ
NSQ
HEX
Pre-myoblasts
Myoblasts
Pre-osteoblasts
Osteoblasts
Chondrocytes
Fibroblasts
0 5 10 15
Cell area x 1000 (um²)
0 4 8
Nucleus area x 100 (um²)
0 5 10
pFAK/FAK intensity
0 2 4 6 8
Actin integrated intensity x 100 (a.u.)
0 20 40
Focal adhesion area (um²)

(caption for figure in previous page) **Supplementary Figure 2. Conventional measurements of cells and focal adhesions reveal cell type specific changes induced by nanotopography.** Histograms cell area, nucleus area, pFAK/FAK integrated intensity ratio, actin integrated intensity and focal adhesion area all cell type and topography combinations. Nanotopographies are color coded, with FLAT denoted in pink, SQ denoted in purple, NSQ denoted in blue and HEX denoted in green.

Supplementary Note 1

Cells manifested qualitative differences after 2 days. Pre-myoblasts and myoblasts were highly elongated on FLAT but were more irregularly shaped with highly discrete and large focal adhesions on SQ. Pre-osteoblasts and osteoblasts had very prominent actin stress fibers and large adhesions regardless of topography. Chondrocytes generally had low pFAK expression, weak stress fiber formation, and stellate and irregular shapes, particularly on SQ and NSQ. Focal adhesions were largest in osteoblastic cells on NSQ and SQ. Fibroblasts had very diffuse focal adhesions and were small and elongated on FLAT and SQ, while those on NSQ and HEX were larger and with more aligned actin stress fibers.

Statistically significant differences were also noted. NSQ significantly increased cell area relative to FLAT. Changes in pFAK/FAK intensity ratios showed cell type dependence: chondrocytes cultured on SQ, pre-osteoblasts cultured on HEX, and fibroblasts on HEX showed the highest pFAK activation compared to the FLAT control. Generally, both pre-myoblasts and myoblasts showed low pFAK activation compared to all cell types. Actin intensity changed most markedly in chondrocytes cultured on HEX, showing higher variance and higher intensity values compared with the FLAT control. Pre-osteoblasts and osteoblasts on NSQ and HEX developed focal adhesions with the largest area compared to all cell types.

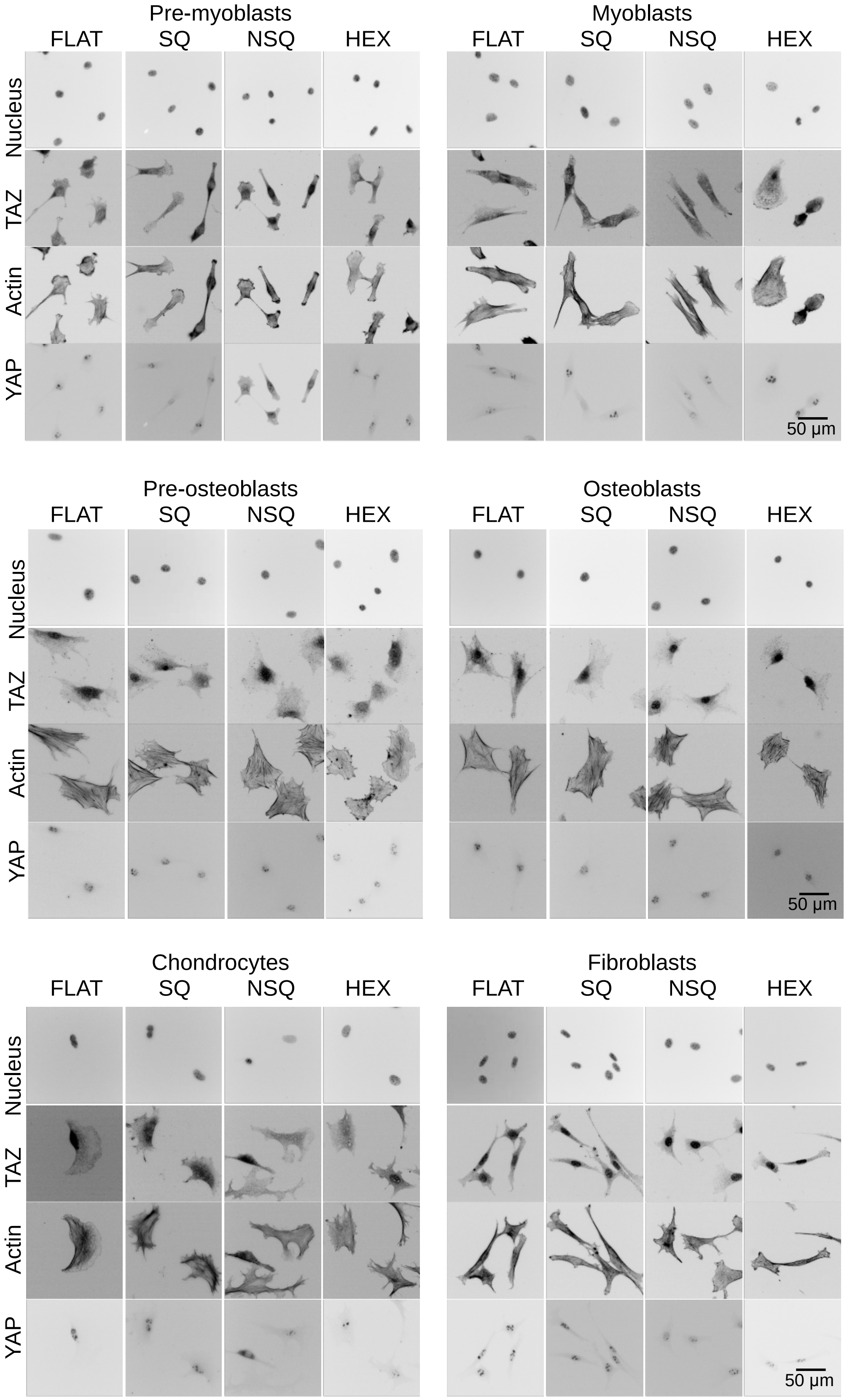

Supplementary Figure 3. Representative images of cellular YAP/TAZ expression and localization in response to nanotopography. Cells were stained using antibodies against the nucleus, actin cytoskeleton, and the mechanosensors YAP and TAZ then imaged after 2 days in culture on the nanotopographies. Scale bar = 50 µm.

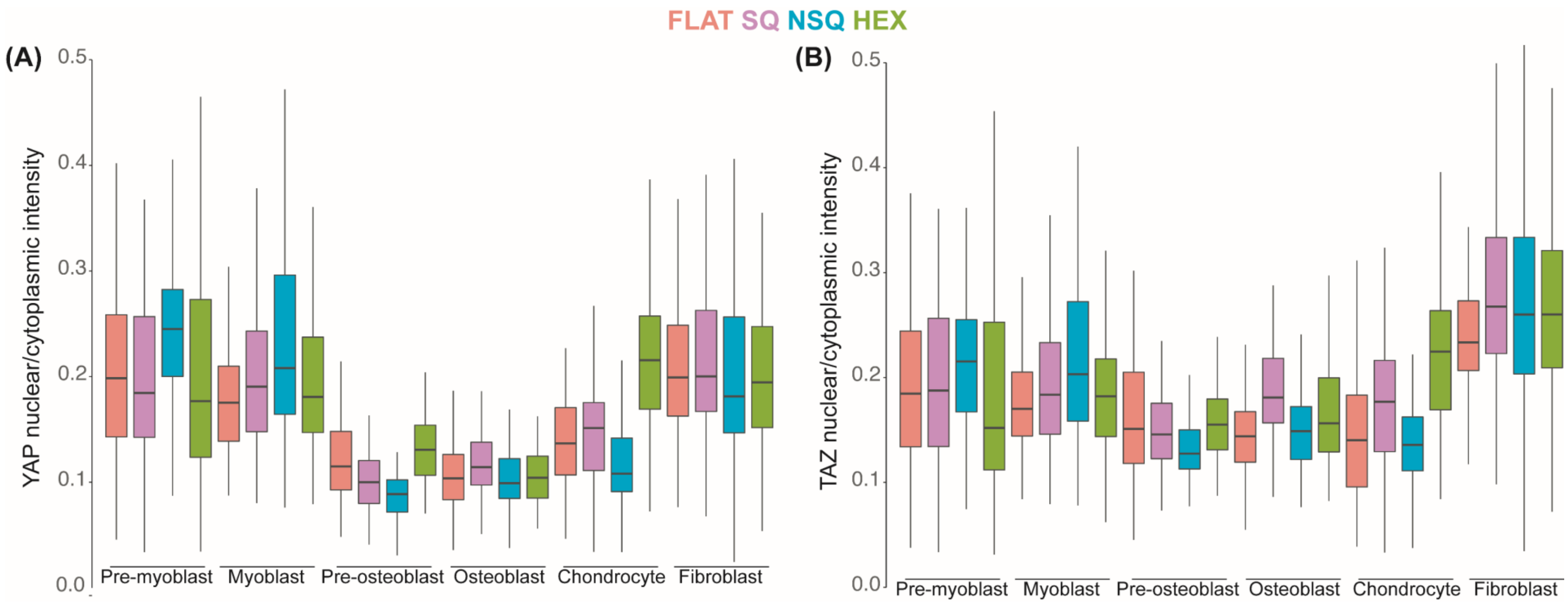

Supplementary Figure 4. Quantification of YAP/TAZ nuclear localization changed by nanotopography. The ratio of integrated intensity in the nucleus normalized to total intensity in the cell of (A) YAP or (B) TAZ are shown here. Changes in the nuclear/cytoplasmic intensities of YAP or TAZ indicate changes in mechanosignaling induced by nanotopographical cues. The effects of nanotopography on YAP or TAZ nuclear translocation vary according to the cell type. Relative to its FLAT controls, NSQ induced higher whereas SQ showed lower nuclear translocation of YAP and TAZ on myoblastic and osteoblastic cells, respectively. Boxplots show median and interquartile range, with minima and maxima at the whiskers. Nanotopographies are color coded, with FLAT denoted in pink, SQ denoted in purple, NSQ denoted in blue and HEX denoted in green.

Supplementary Table 1. Descriptors/features measured by various modules on CellProfiler. Descriptions from the CellProfiler documentation[1], Kumar et al.[2], Vega et al.[3], and Huang et al[4].

**Area and Shape**		
Area	Total number of pixels in each object.	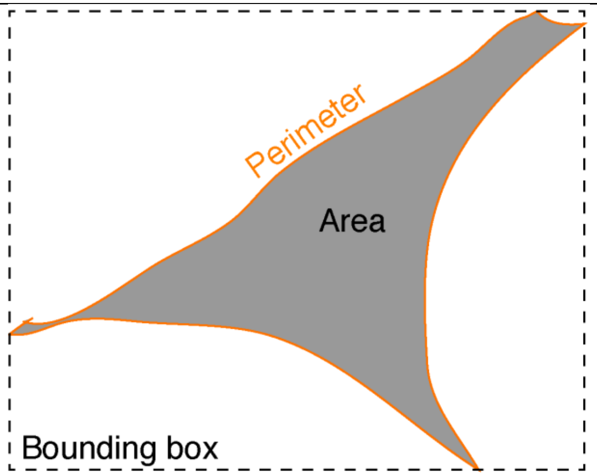
Perimeter	Total number of pixels on the boundary of each object.	
Form Factor	Ratio of the area to the perimeter of each object. Ratio of 1 is for a perfect circle.	
Extent	Given the smallest bounding box that can enclose the object, the extent is the ratio of the area of the object by the area of the bounding box. Cells that have fewer protrusions have larger extent values.	
Eccentricity	Ratio of the distance between two foci and the major axis of the ellipse that bounds the cell. Ratio approaching 1 indicates a more elliptical shape.	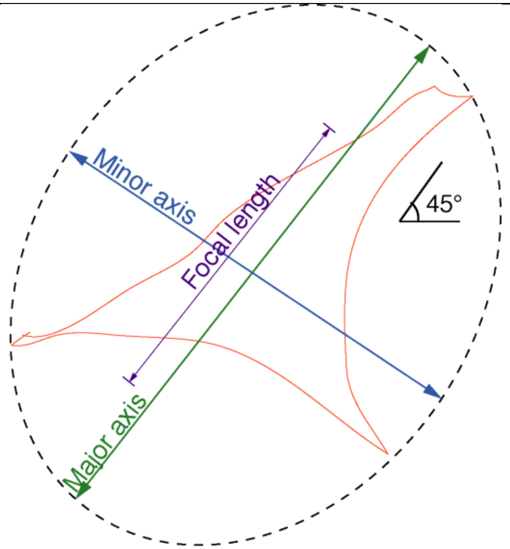
Major Axis Length	Length of the major axis of the ellipse that bounds the cell given in pixels.	
Minor axis length	Length of the minor axis of the ellipse that bounds the cell given in pixels.	
Orientation	Angle between -90 and 90 degrees that indicates the orientation of the major axis of the cell with respect to the horizontal.	

<table>
<tr><td>Maximum Radius</td><td>The greatest distance between any pixel inside the object to the nearest pixel outside of the object.</td><td rowspan="2">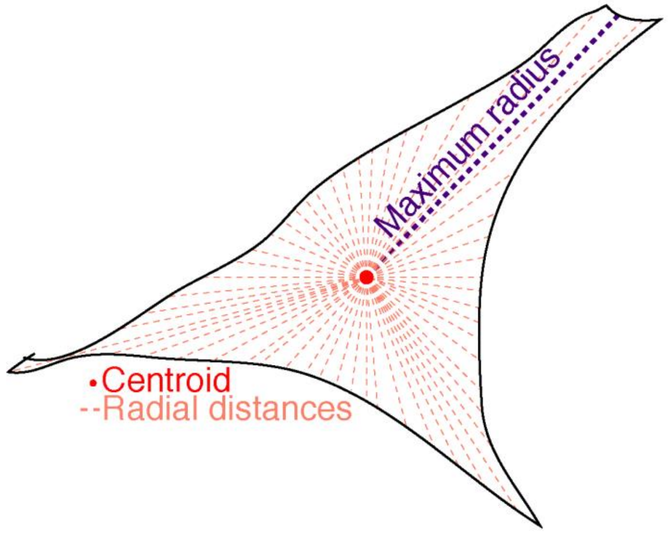
</td></tr>
<tr><td>Compactness</td><td>Ratio of the variance in the radial distance from the centroid and the area of the object.</td></tr>
<tr><td>Maximum Feret Diameter</td><td>The maximum distance between two parallel lines tangent to opposite sides of the object.</td><td rowspan="2">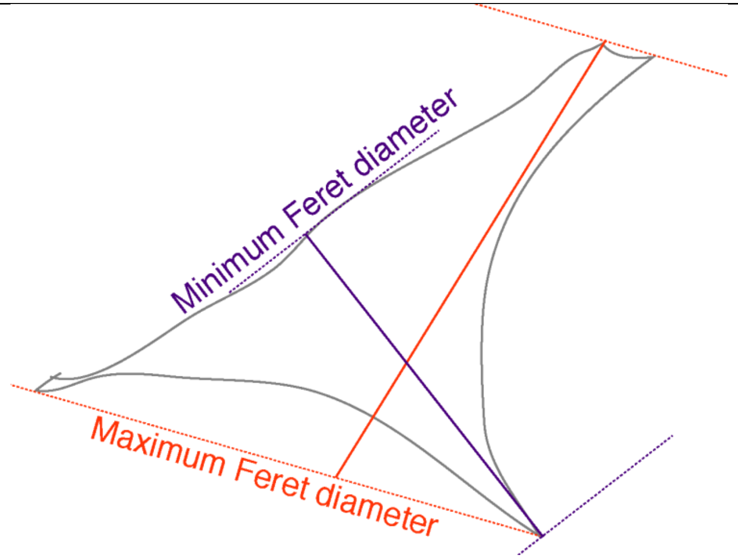
</td></tr>
<tr><td>Minimum Feret Diameter</td><td>The minimum distance between two parallel lines tangent to opposite sides of the object.</td></tr>
</table>

Solidity	Measure of how many holes or concave boundaries in each object. Measured as area/convex hull area. The convex hull area is fitted onto the object by drawing a straight line across straight or concave areas of the cell but expanding outward along object protrusions. Ratio of 1 indicates an object without any concavities or indentations, while a ratio less than 1 indicates an object with holes or an irregular boundary. Ratio of Area to convex hull area.	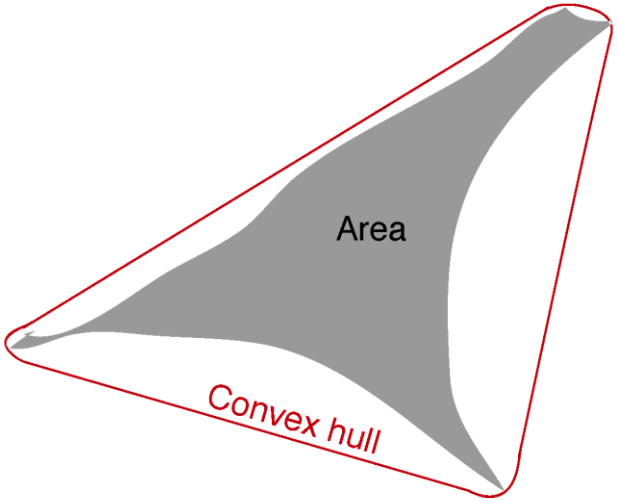
Zernike shape features	Set of polynomial coefficients used to describe cell shape with increasing detail. The smallest circle that encloses the object is used to calculate Zernike features. Cells that are more closely related to the Zernike polynomial in question are reflected in a higher value.	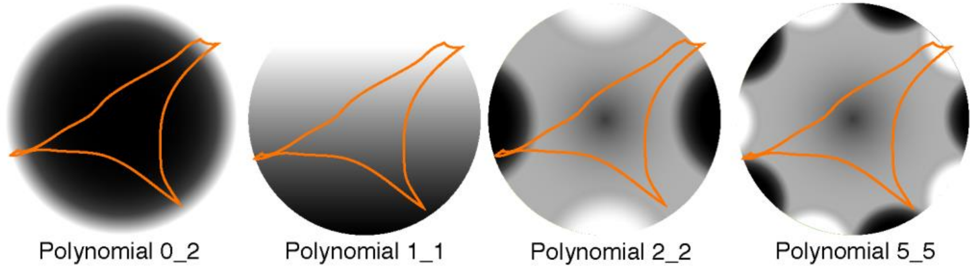<br>Cell is low in Zernike coefficients for polynomials 0_2 and 2_2 but high for polynomial 1_1 and 5_5

Intensity		
Integrated Intensity	Total intensity values of pixels within a specified object. (e.g. measurement of total intensity of focal adhesions within a single cell)	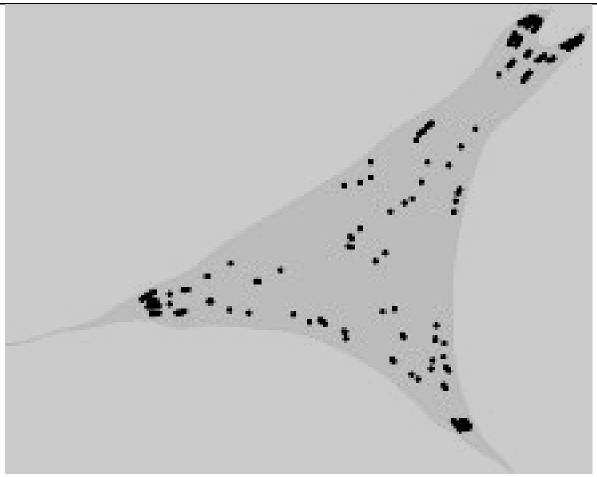
Integrated Edge Intensity	The sum of pixel intensities along the perimeter of the object. (e.g. measurement of total intensity of focal adhesions on the perimeter of the object)	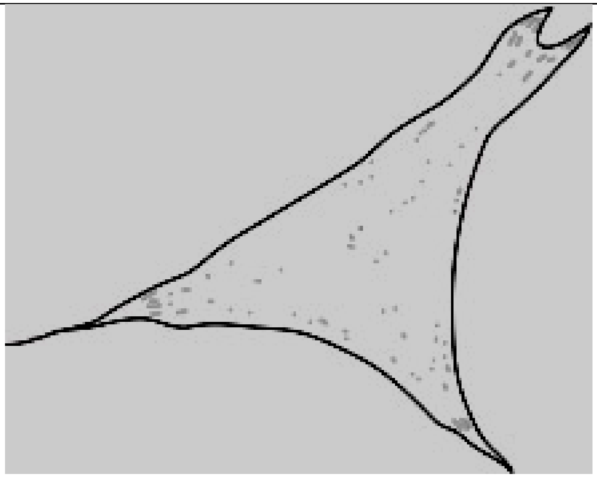
Fraction at Distance (FracAtD)	The ratio of the stain in object to the total stain within a given radius (measured from the cell nucleus).	

<table>
<tr><td>Mean Fractional Intensity (MeanFrac)</td><td>The mean fractional intensity at a specified radius (with the center defined as the nucleus).</td><td rowspan="2">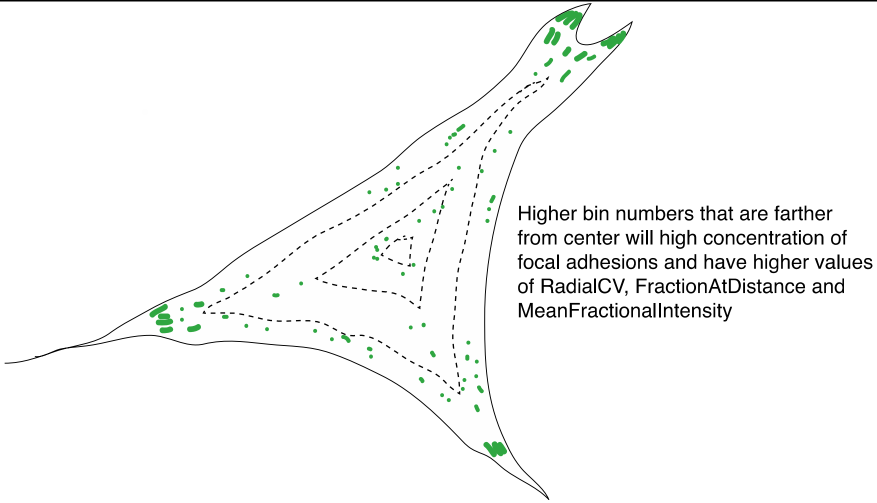
</td></tr>
<tr><td>Radial coefficient of variation (RadialCV)</td><td>Coefficient of variation of intensity within a ring (with the center defined as the nucleus).</td></tr>
<tr><td>Zernike Magnitude</td><td>Characterizes the distribution of intensity across an object from its center described by Zernike polynomials. Higher Zernike polynomials describe the object with higher detail. Values obtained are correlation coefficients between the intensity distribution within the object and the given Zernike polynomial.</td><td rowspan="2">Distribution of intensity according to Zernike polynomials
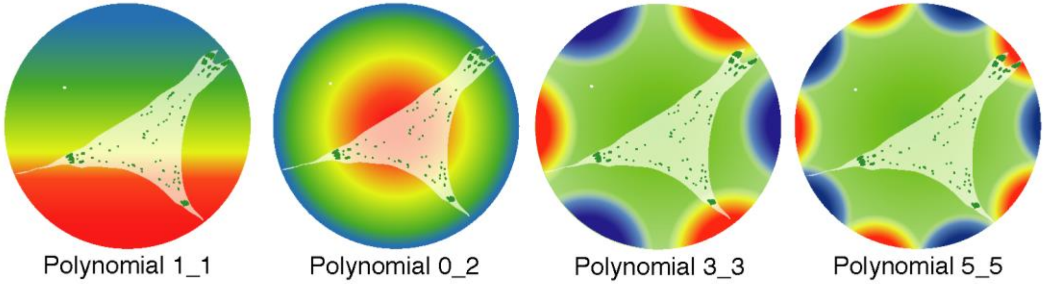

Zernike coefficents for focal adhesions is low for 1_1 but high for 0_2, 3_3 and 5_5</td></tr>
<tr><td>Zernike Phase</td><td>Provides information about the orientation of the Zernike magnitude measurement.</td></tr>
</table>

**Haralick texture features**<br>The Haralick texture features measure pixel frequency information in an object and this information is summarized in each Haralick measure.	
Angular Second Moment	Measures object homogeneity in pixel gray levels. A high value indicates very similar pixels within the vicinity. Based on Haralick's measure, H1
Contrast	Measures the local intensity variations between a reference pixel and its neighbors. A high values indicate large differences in intensity. Based on Haralick's measure, H2
Correlation	Indicates the linearity of the object. A high value is obtained when the object contains linear structures. Based on Haralick's measure, H3
Variance (Sum of Squares)	Measures heterogeneity of pixel gray levels in each object. Based on Haralick's measure, H4
Inverse Difference Moment	Measures local homogeneity. A high value indicates uniform local gray levels. Based on Haralick's measure, H5
Sum Variance	Summation of variance measurements across each object, which gives the distribution of size in grains measured by granularity. Based on Haralick's measure, H7
Sum Entropy	Summation of entropy measurements across each object, which is low for uniform objects. Based on Haralick's measure, H8
Entropy	Measures the degree of randomness in the object. Higher entropy values occur when there is more heterogeneity in neighboring pixels. Based on Haralick's measure, H9
Difference Variance	Difference in variance measurements across each object. Based on Haralick's measure, H10
Difference Entropy	Difference in entropy measurements across each object, which is high for uniform objects. Based on Haralick's measure, H11
Information Measure of Correlation 1	Ratio of Entropy to inverse difference moment/local homogeneity of the object. Measure of the uncertainty in correlation measurements. Based on Haralick's measure, H12
Information Measure of Correlation 2	Value related to the square root of entropy. Measure of the uncertainty in correlation measurements. Based on Haralick's measure, H13

Granularity	Measures the grain size. High values indicate the coarseness of the texture.
**Non-Haralick textures**	
Gabor factor	Measures correlation between bands of intensities. A higher correlation of pattern of intensity results in a higher Gabor feature measurement (e.g. parallel actin stress fibers lead to a higher Gabor factor value)

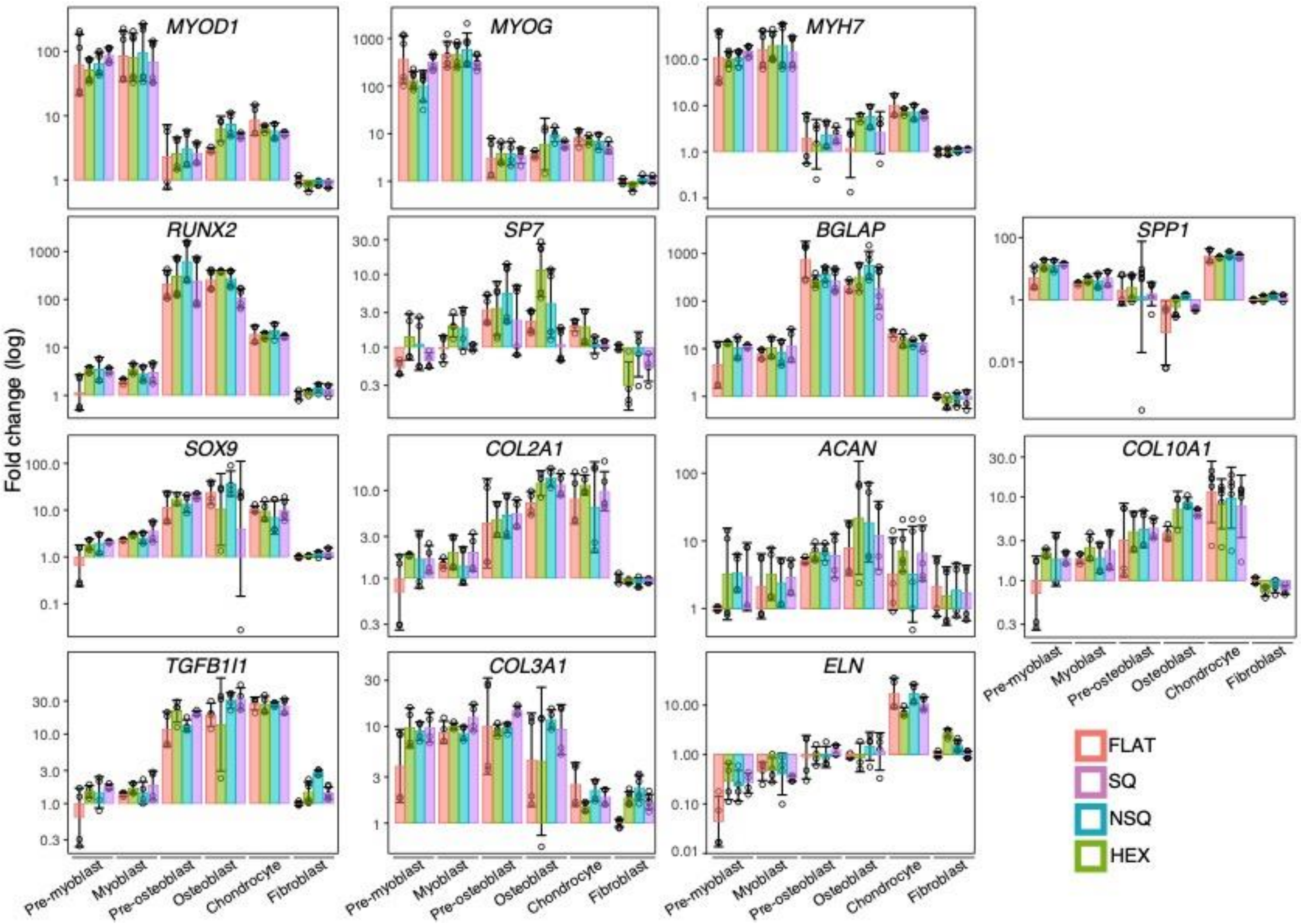

Supplementary Figure 5. Measurement of musculoskeletal marker expression on all combinations of cell type and topography. Quantitative polymerase chain reaction (qPCR) was used to determine the changes in gene expression across 14 different musculoskeletal genes. Fold change in expression of each gene expression were calculated with respect to fibroblasts on Flat controls (n>4 from 2 independent experiments). Nanotopographies are color coded, with FLAT denoted in pink, SQ denoted in purple, NSQ denoted in blue and HEX denoted in green. Data are presented as mean ± standard deviation, with individual data points presented as open faced circles.

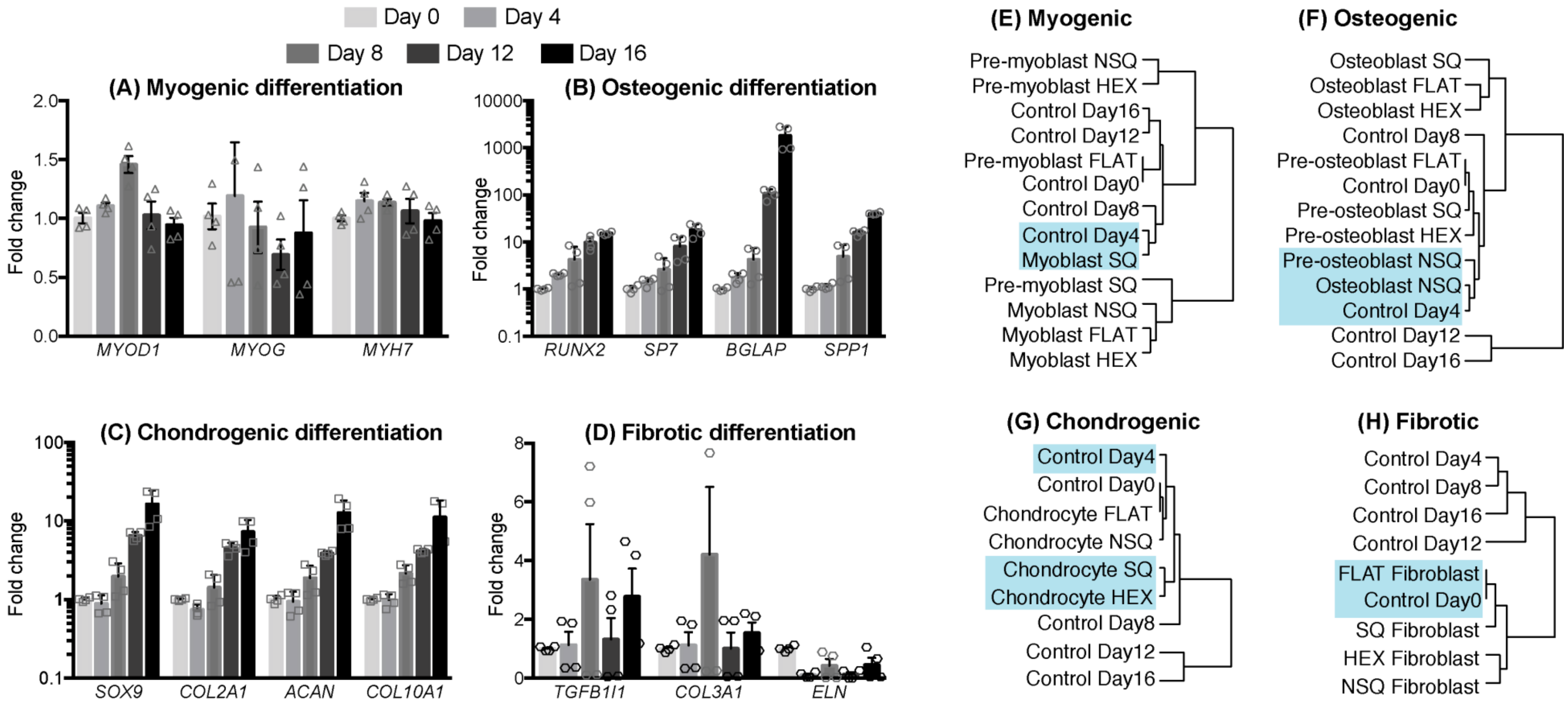

Supplementary Figure 6. Comparison of gene expression between cells stimulated by nanotopography and biochemical inducers of differentiation. (A-D) Control experiments were performed by differentiating pre-myoblasts, pre-osteoblasts, chondrocytes and fibroblasts grown on standard tissue culture polystyrene with established biochemical inducers. Thereafter, gene expression from the control experiments were measured at different timepoints. Day 0 indicates cells undifferentiated cells. (A) Myogenic, (B) osteogenic, (C) chondrogenic and (D) fibrotic differentiation were measured across multiple timepoints (n=6, 2 independent experiments). Data are presented as mean ± standard deviation, with individual data points presented as open faced circles. (E-H) Hierarchical clustering of average gene expression data. Dendrogram shows relationship in gene expression between biochemically-differentiated controls and nanotopographically-stimulated cells. Notable similarities in biochemically-driven differentiation (control) and nanotopography-induced gene expression are highlighted in blue. Gene expression from cells on nanotopography were measured after 7 days of culture, and are originally presented in Figure 3 of the main text.

Supplementary Table 2. Exact p values denoting statistically significant comparisons of gene expression. Gene expression measurements presented in Figure 3 were compared using one-way ANOVA with Tukey's post-hoc test for pairwise comparison.

Gene	Pairwise comparison	p value
MYOD1	Pre-myoblast FLAT vs Pre-myoblast SQ	0.0078
MYOG	Pre-myoblast FLAT vs Pre-myoblast SQ	0.0327
MYH7	Pre-myoblast FLAT vs Pre-myoblast SQ	0.030
RUNX2	Pre-osteoblast FLAT vs Pre-osteoblast SQ	0.0001
RUNX2	Pre-osteoblast FLAT vs Pre-osteoblast NSQ	0.0001
RUNX2	Pre-osteoblast FLAT vs Pre-osteoblast HEX	0.0001
RUNX2	Osteoblast FLAT vs Osteoblast SQ	0.0001
SP7	Pre-osteoblast FLAT vs Pre-osteoblast NSQ	<0.0001
SP7	Osteoblast FLAT vs Osteoblast NSQ	<0.0001
SP7	Osteoblast FLAT vs Osteoblast HEX	<0.0001
BGLAP	Pre-osteoblast FLAT vs Pre-osteoblast HEX	<0.0001
BGLAP	Osteoblast FLAT vs Osteoblast NSQ	<0.0001
SPP1	Pre-osteoblast FLAT vs Pre-osteoblast SQ	0.0377
SPP1	Pre-osteoblast FLAT vs Pre-osteoblast NSQ	<0.0001
SPP1	Pre-osteoblast FLAT vs Pre-osteoblast HEX	<0.0001
SPP1	Osteoblast FLAT vs Osteoblast NSQ	<0.0001
COL2A1	Chondrocyte FLAT vs Chondrocyte HEX	0.003
ACAN	Chondrocyte FLAT vs Chondrocyte HEX	0.0001
TGFB1I1	Fibroblast FLAT vs Fibroblast NSQ	0.0037
COL3A1	Fibroblast FLAT vs Fibroblast SQ	0.0032
COL3A1	Fibroblast FLAT vs Fibroblast NSQ	<0.0001
COL3A1	Fibroblast FLAT vs Fibroblast HEX	0.0006
ELN	Fibroblast FLAT vs Fibroblast HEX	0.0003

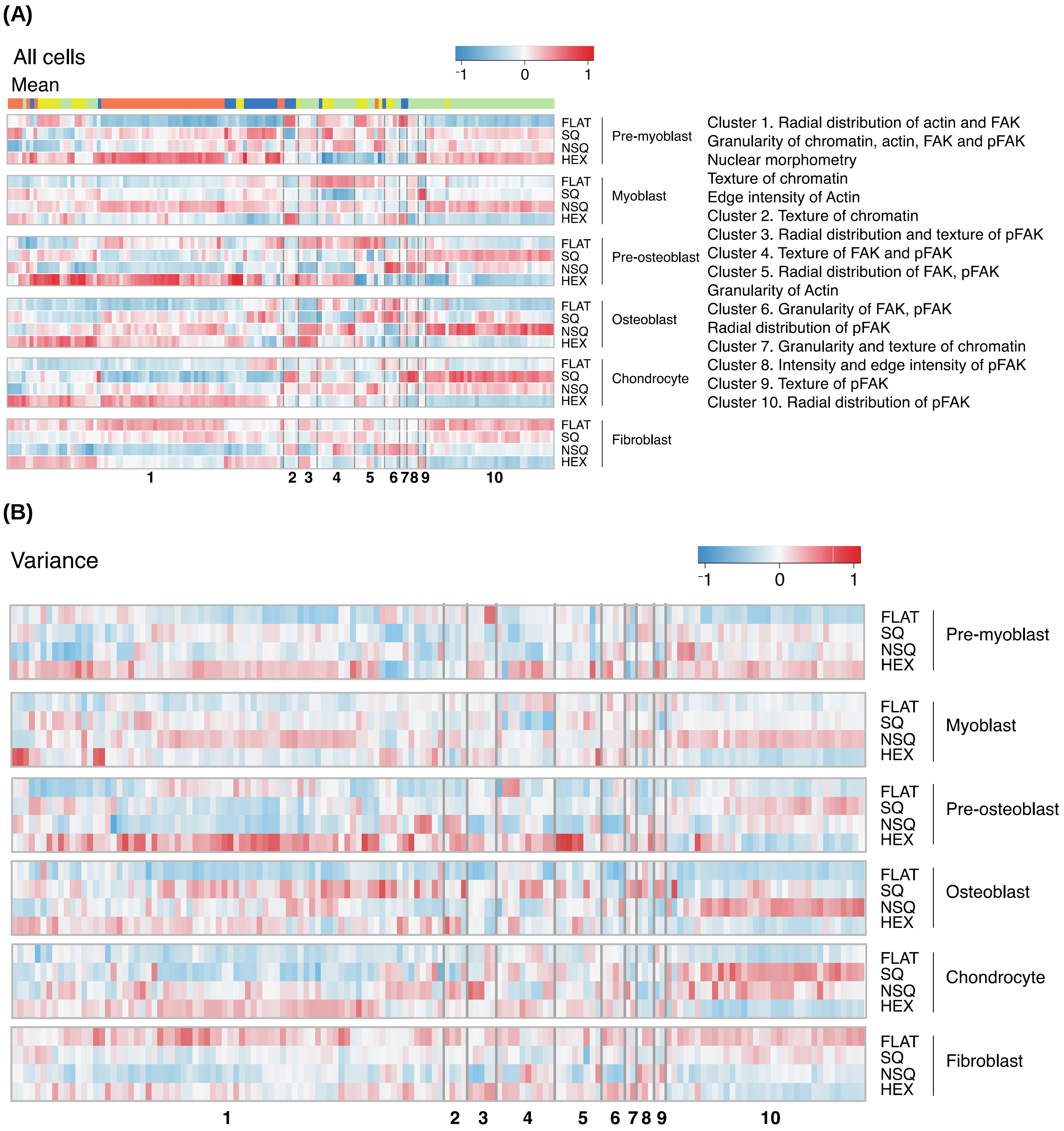

Supplementary Figure 7. Hierarchical clustering of the morphome across nanotopographies. (A) Mean measurements and (B) variance of the morphome across nanotopographies were clustered. For visualization, each morphome feature was normalized to have a mean value of 0 and standard deviation of 1 across all datapoints. Features included were significantly varied across cell types ($p < 0.05$ using one-way ANOVA). The complete list of morphome features used is found in Supplementary Data 3.

Supplementary Table 3. Accuracy of classifying the morphome into different cell types using Bayesian logistic regression with a leave-one-out scheme. Bayesian logistic regression models were trained by holding out from the entire dataset a cell type from one independent experiment. The held-out dataset was used as test data to determine classification accuracy. We present here classification accuracy from each held-out experiment, and the mean ± standard deviation of classification accuracy from two independent experiments. Essentially, we show that the morphome contains sufficient information to predict cell type outside of the dataset.

Cell type	Experiment 1 (%)	Experiment 2 (%)	Mean ± standard deviation (%)
Pre-myoblast	56.0	45.2	50.6 ± 7.6
Myoblast	45.3	59.5	52.4 ± 10.1
Pre-osteoblast	60.1	66.7	63.4 ± 4.7
Osteoblast	60.1	70.1	65.1 ± 7.0
Chondrocyte	40.0	33.9	36.8 ± 4.3
Fibroblast	24.7	35.9	30.3 ± 7.8
Random	-	-	16.7

Supplementary Table 4. Accuracy of classifying the morphome into different cell types using Bayesian logistic regression. Bayesian logistic regression models were trained using a 60% of the entire dataset. The remaining 40% of the dataset was used to test accuracy of cell type classification.

Cell type	Classification accuracy (%)
Pre-myoblast	92.6
Myoblast	89.3
Pre-osteoblast	95.7
Osteoblast	95.2
Chondrocyte	97.2
Fibroblast	97.4
Random	16.7

Supplementary Note 2

The leave-one-out scheme used to measure classification accuracy in Supplementary Table 3 was used to prevent overfitting the data to experimental variation (e.g. in background staining). For this new classification scheme, we iteratively removed from the entire dataset a cell type from one independent experiment (the held-out dataset, e.g. Chondrocyte from the first independent experiment). The remaining dataset was used to train a Bayesian logistic regression model (details in Supplementary Methods) to classify a cell type using the morphome. The left-out dataset was then used as test data to measure the accuracy of classification using the morphome. That is, in the training set we do not see examples of the cell types from a biological experiment, but we exploit the availability of other cell types to avoid overfitting to any individual experimental setup. We averaged the classification accuracy of each class from two independent experiments and present the average ± standard deviation. In this manner, we obtained a measure of the predictive quality of the morphome that does not conflate information about the specific cell type in question from independent experiments. Essentially this leave-one-out scheme allows us to predict outside of the dataset, an important task supporting the claim that the morphome contains sufficient information to classify cell types.

Overall, we showed that classification of cells using the morphome still outperformed random classification (Supplementary Table 3). This is similar to what we observed when we use the conventional machine learning method that separates the dataset into train and test sets without regard to experimental variation (Supplementary Table 4). Both these results align with what we observed in Figure 5b, where we demonstrated that prediction of gene expression outside of the training dataset suffers most with removal of the cell type related to the gene expression of interest.

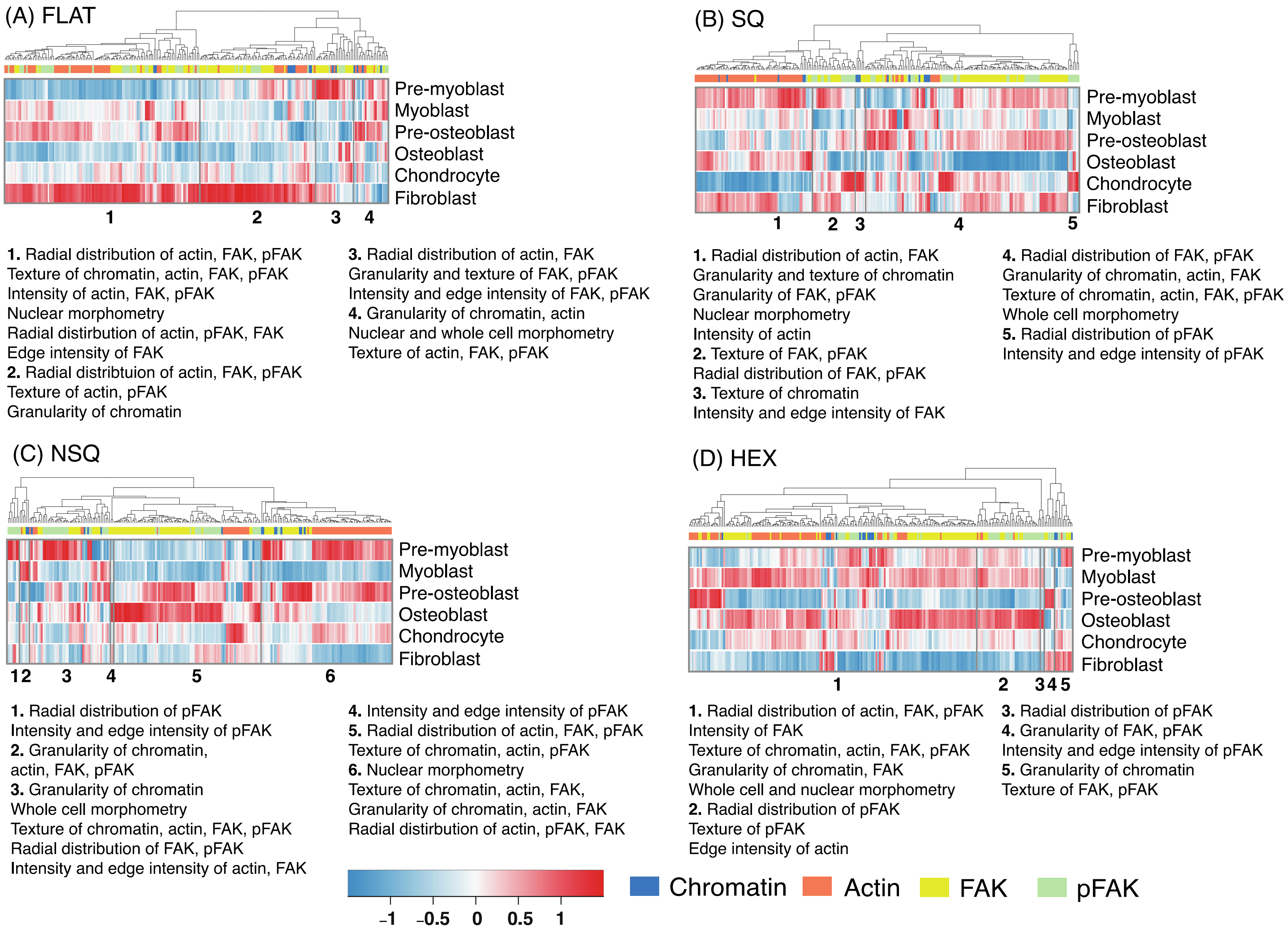
(A) FLAT
Pre-myoblast
Myoblast
Pre-osteoblast
Osteoblast
Chondrocyte
Fibroblast
1 2 3 4
1. Radial distribution of actin, FAK, pFAK
Texture of chromatin, actin, FAK, pFAK
Intensity of actin, FAK, pFAK
Nuclear morphometry
Radial distirbution of actin, pFAK, FAK
Edge intensity of FAK
2. Radial distribtuion of actin, FAK, pFAK
Texture of actin, pFAK
Granularity of chromatin
3. Radial distribution of actin, FAK
Granularity and texture of FAK, pFAK
Intensity and edge intensity of FAK, pFAK
4. Granularity of chromatin, actin
Nuclear and whole cell morphometry
Texture of actin, FAK, pFAK
(B) SQ
Pre-myoblast
Myoblast
Pre-osteoblast
Osteoblast
Chondrocyte
Fibroblast
1 2 3 4 5
1. Radial distribution of actin, FAK
Granularity and texture of chromatin
Granularity of FAK, pFAK
Nuclear morphometry
Intensity of actin
2. Texture of FAK, pFAK
Radial distribution of FAK, pFAK
3. Texture of chromatin
Intensity and edge intensity of FAK
4. Radial distribution of FAK, pFAK
Granularity of chromatin, actin, FAK
Texture of chromatin, actin, FAK, pFAK
Whole cell morphometry
5. Radial distribution of pFAK
Intensity and edge intensity of pFAK
(C) NSQ
Pre-myoblast
Myoblast
Pre-osteoblast
Osteoblast
Chondrocyte
Fibroblast
1 2 3 4 5 6
1. Radial distribution of pFAK
Intensity and edge intensity of pFAK
2. Granularity of chromatin,
actin, FAK, pFAK
3. Granularity of chromatin
Whole cell morphometry
Texture of chromatin, actin, FAK, pFAK
Radial distribution of FAK, pFAK
Intensity and edge intensity of actin, FAK
4. Intensity and edge intensity of pFAK
5. Radial distribution of actin, FAK, pFAK
Texture of chromatin, actin, pFAK
6. Nuclear morphometry
Texture of chromatin, actin, FAK,
Granularity of chromatin, actin, FAK
Radial distirbution of actin, pFAK, FAK
(D) HEX
Pre-myoblast
Myoblast
Pre-osteoblast
Osteoblast
Chondrocyte
Fibroblast
1 2 3 4 5
1. Radial distribution of actin, FAK, pFAK
Intensity of FAK
Texture of chromatin, actin, FAK, pFAK
Granularity of chromatin, FAK
Whole cell and nuclear morphometry
2. Radial distribution of pFAK
Texture of pFAK
Edge intensity of actin
3. Radial distribution of pFAK
4. Granularity of FAK, pFAK
Intensity and edge intensity of pFAK
5. Granularity of chromatin
Texture of FAK, pFAK
–1 –0.5 0 0.5 1
Chromatin
Actin
FAK
pFAK

(caption for figure in previous page) **Supplementary Figure 8. Morphome separated by nanotopographies clustered characteristically across each cell type.** (A-D) Hierarchical clustering of the morphome separated by nanotopography. Cell type specific morphome features are made up of 15 chromatin, 73 actin, 88 FAK and 56 pFAK features. Each morphome feature was mean centered and normalized to standard deviation per cell type to result in mean = 0 and standard deviation = 1. The total number of cells analysed per nanotopography type are: (A) n=1251 for FLAT; (B) n=1215 for SQ; (C) n=1293 for NSQ; (D) n=1180 for HEX obtained across 2 independent experiments. The colour and intensity of each tile represents the average value of the feature for the specified cell type and nanotopography.

Supplementary Note 3

On FLAT, pre-myoblasts and osteoblasts showed predominantly low average values for cell-type specific features except for morphometric and texture measurements (Supplementary Figure 8, cluster 4). This was reversed in fibroblasts, with particularly high average values of FAK and actin radial distribution. Pre-myoblasts showed high average values for actin radial distribution and intensity, nuclear morphometry, and chromatin textures on SQ (cluster 1). Chondrocytes showed the opposite profile for the same features. Average values for FAK measurements were high on pre-myoblasts and pre-osteoblasts and low on osteoblasts (cluster 4). On NSQ, pre-osteoblasts and osteoblasts showed markedly high average values of FAK radial distribution (cluster 5), relative to other cell types while pre-myoblasts and myoblasts cells showed low average values of FAK radial distribution. Pre-myoblasts and myoblasts cells showed the opposite for the same features. While pre-osteoblasts and myoblasts demonstrated high average values of actin radial distribution and textures (cluster 6), osteoblasts and myoblasts showed low average values for the same features. Cell type-specific features were mostly high on pre-myoblasts, myoblasts and osteoblasts on HEX. On the other hand, fibroblasts and pre-osteoblasts showed low average values for the cell-type specific features. Surprisingly, chondrocytes exhibited moderate values (close to the mean) of the cell type-specific features. The cell type-specific hierarchical clusters show low correlation (Supplementary Table 5).

Supplementary Table 5. Correlation coefficient of the morphome hierarchically clustered by nanotopography.

	All topographies	FLAT	SQ	NSQ	HEX
All topographies	1				
FLAT	0.356	1			
SQ	0.389	0.140	1		
NSQ	0.564	0.276	0.328	1	
HEX	0.458	0.194	0.229	0.451	1

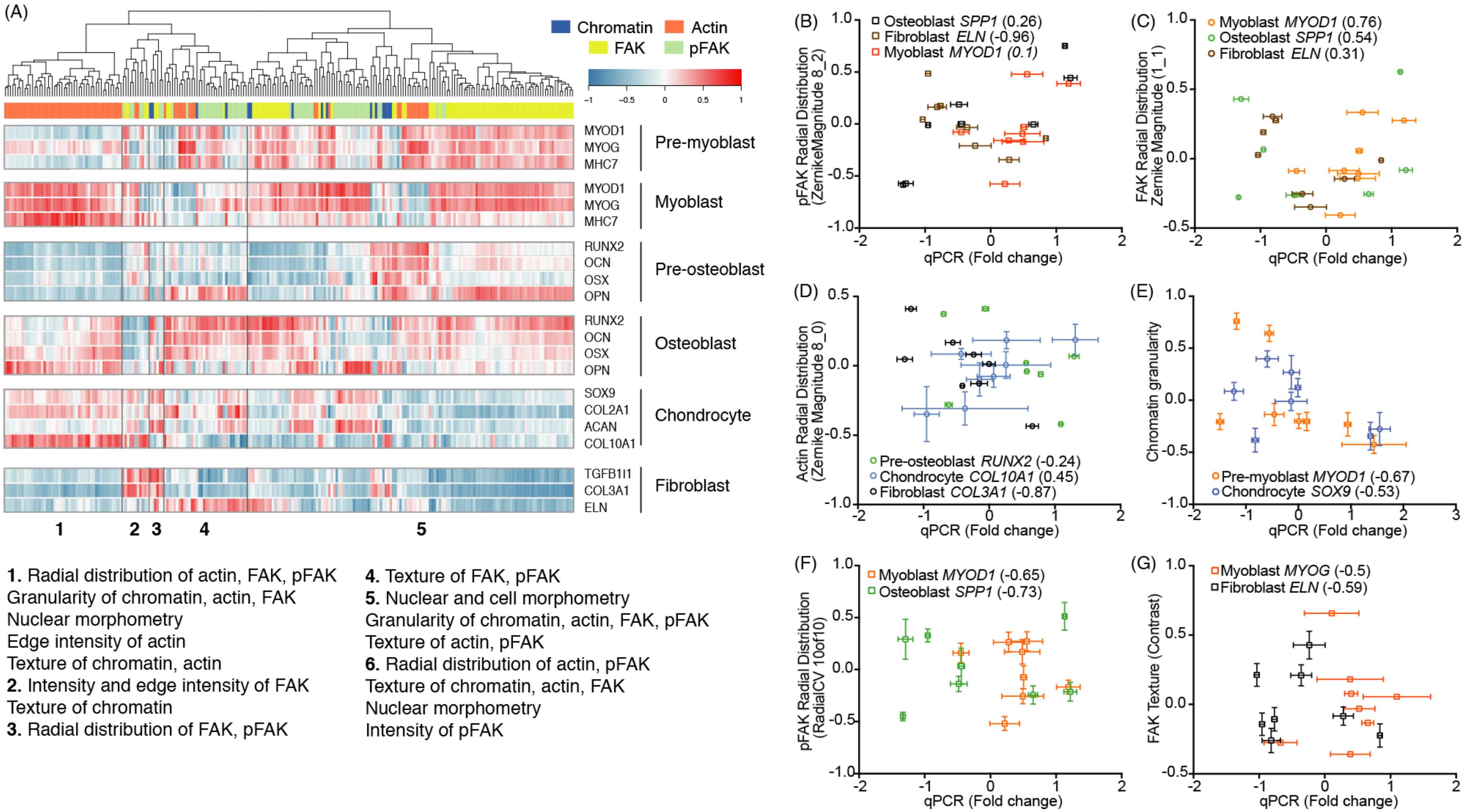

(A)
Chromatin
Actin
FAK
pFAK
-1
-0.5
0
0.5
1
MYOD1
MYOG
MHC7
Pre-myoblast
MYOD1
MYOG
MHC7
Myoblast
RUNX2
OCN
OSX
OPN
Pre-osteoblast
RUNX2
OCN
OSX
OPN
Osteoblast
SOX9
COL2A1
ACAN
COL10A1
Chondrocyte
TGFB1I1
COL3A1
ELN
Fibroblast
1
2
3
4
5
1. Radial distribution of actin, FAK, pFAK
Granularity of chromatin, actin, FAK
Nuclear morphometry
Edge intensity of actin
Texture of chromatin, actin
2. Intensity and edge intensity of FAK
Texture of chromatin
3. Radial distribution of FAK, pFAK
4. Texture of FAK, pFAK
5. Nuclear and cell morphometry
Granularity of chromatin, actin, FAK, pFAK
Texture of actin, pFAK
6. Radial distribution of actin, pFAK
Texture of chromatin, actin, FAK
Nuclear morphometry
Intensity of pFAK
(B)
Osteoblast SPP1 (0.26)
Fibroblast ELN (-0.96)
Myoblast MYOD1 (0.1)
pFAK Radial Distribution (ZernikeMagnitude 8_2)
qPCR (Fold change)
(C)
Myoblast MYOD1 (0.76)
Osteoblast SPP1 (0.54)
Fibroblast ELN (0.31)
FAK Radial Distribution Zernike Magnitude (1_1)
qPCR (Fold change)
(D)
Pre-osteoblast RUNX2 (-0.24)
Chondrocyte COL10A1 (0.45)
Fibroblast COL3A1 (-0.87)
Actin Radial Distribution (Zernike Magnitude 8_0)
qPCR (Fold change)
(E)
Pre-myoblast MYOD1 (-0.67)
Chondrocyte SOX9 (-0.53)
Chromatin granularity
qPCR (Fold change)
(F)
Myoblast MYOD1 (-0.65)
Osteoblast SPP1 (-0.73)
pFAK Radial Distribution (RadialCV 10of10)
qPCR (Fold change)
(G)
Myoblast MYOG (-0.5)
Fibroblast ELN (-0.59)
FAK Texture (Contrast)
qPCR (Fold change)

(caption for figure in previous page) **Supplementary Figure 9. Spearman correlation between the changes in morphome and changes in gene expression resulting from nanotopography.** Spearman correlation coefficients were used to measure the similarities in changes between morphome and gene expression across nanotopographies. (A) Hierarchical clustering of the correlation coefficients for each morphome and gene expression pair. The colour and intensity of each tile represents the correlation coefficient magnitude and direction of association between the morphome feature and gene expression. Each feature was mean centered and normalized to standard deviation. (B-G) Scatterplots showing changes in qPCR against individual morphome features. Data are presented as mean (square) ± standard deviation (bars). Spearman rank correlation value between gene expression from specified cell type and the morphome feature (y axis) are presented in parenthesis.

Supplementary Table 6. Mean absolute error (MAE) and coefficient of correlation (R^2) of the predicted gene expression values using the morphome.

	Test set	
Gene	MAE	R2
MYOD1	0.10	0.10
MYOG	0.10	0.10
MYH7	0.10	0.10
RUNX2	0.12	0.12
SP7	0.17	0.17
BGLAP	0.14	0.14
SPP1	0.18	0.18
SOX9	0.15	0.15
COL2A1	0.16	0.15
ACAN	0.12	0.16
COL10A	0.16	0.17
TGFB1I1	0.17	0.17
ELN	0.17	0.21
COL3A1	0.21	0.59
All Genes	0.11	0.72

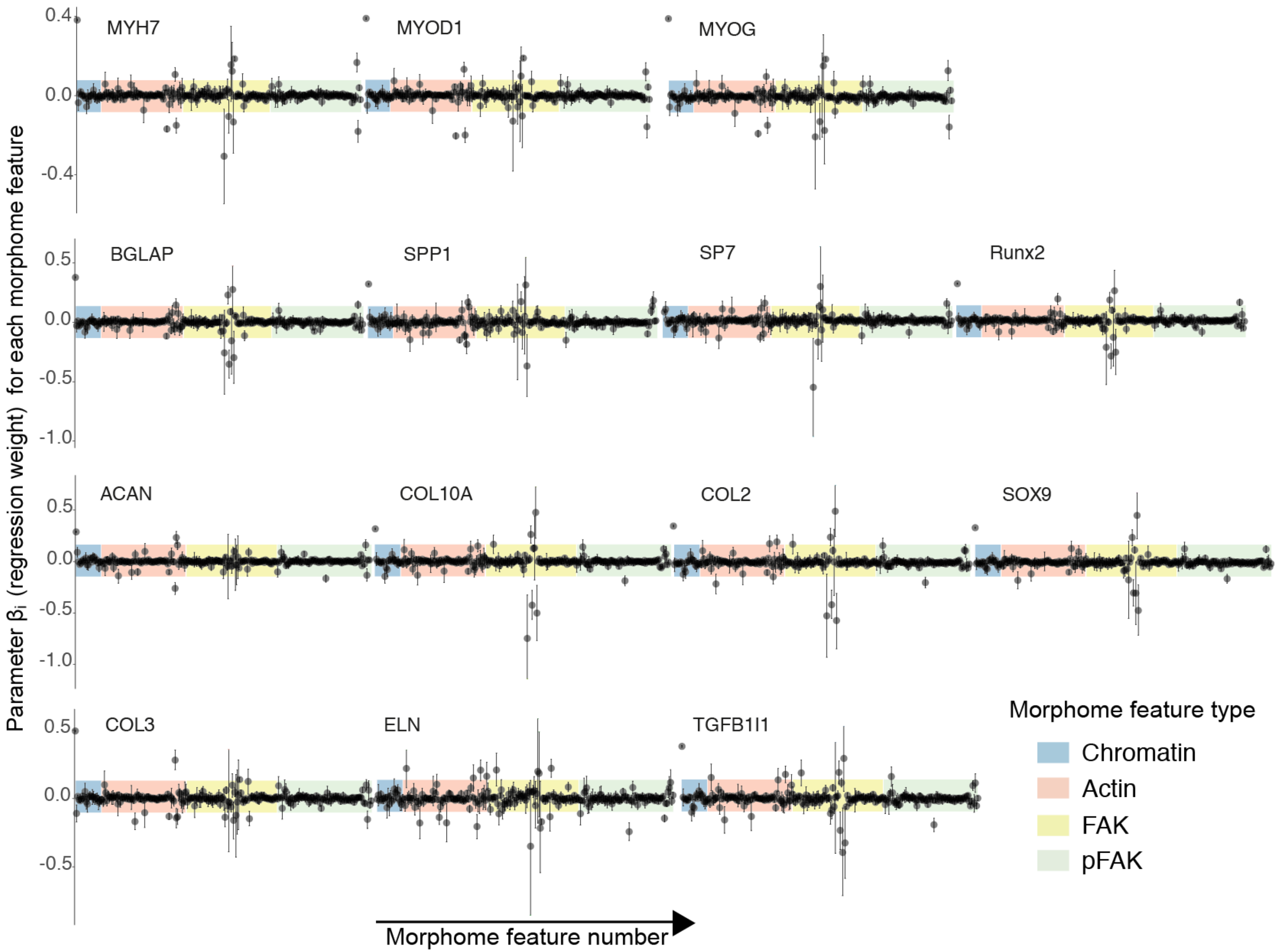

Parameter βi (regression weight) for each morphome feature
MYH7
MYOD1
MYOG
BGLAP
SPP1
SP7
Runx2
ACAN
COL10A
COL2
SOX9
COL3
ELN
TGFB1I1
0.4
0.0
-0.4
0.5
0.0
-0.5
-1.0
Morphome feature number
Morphome feature type
Chromatin
Actin
FAK
pFAK

(caption for figure in previous page) **Supplementary Figure 10. Contribution of each morphome feature to the Bayesian linear regression model.** The parameters β from the linear regression models (i.e. regression weight) weights the contribution of each morphome feature in the Bayesian linear regression model for predicting gene expression. Higher magnitudes of the parameter β indicate larger contribution of the morphome feature in predicting the expression of the specified gene. Data shown are mean (dots) ± high density interval (bars) of β for each morphome feature and gene. The morphome features were color coded to denote chromatin (blue), actin (orange), FAK (yellow) and pFAK (green) measurements. The average value of the model parameter β for each morphome feature and gene is provided in Supplementary Data 5.

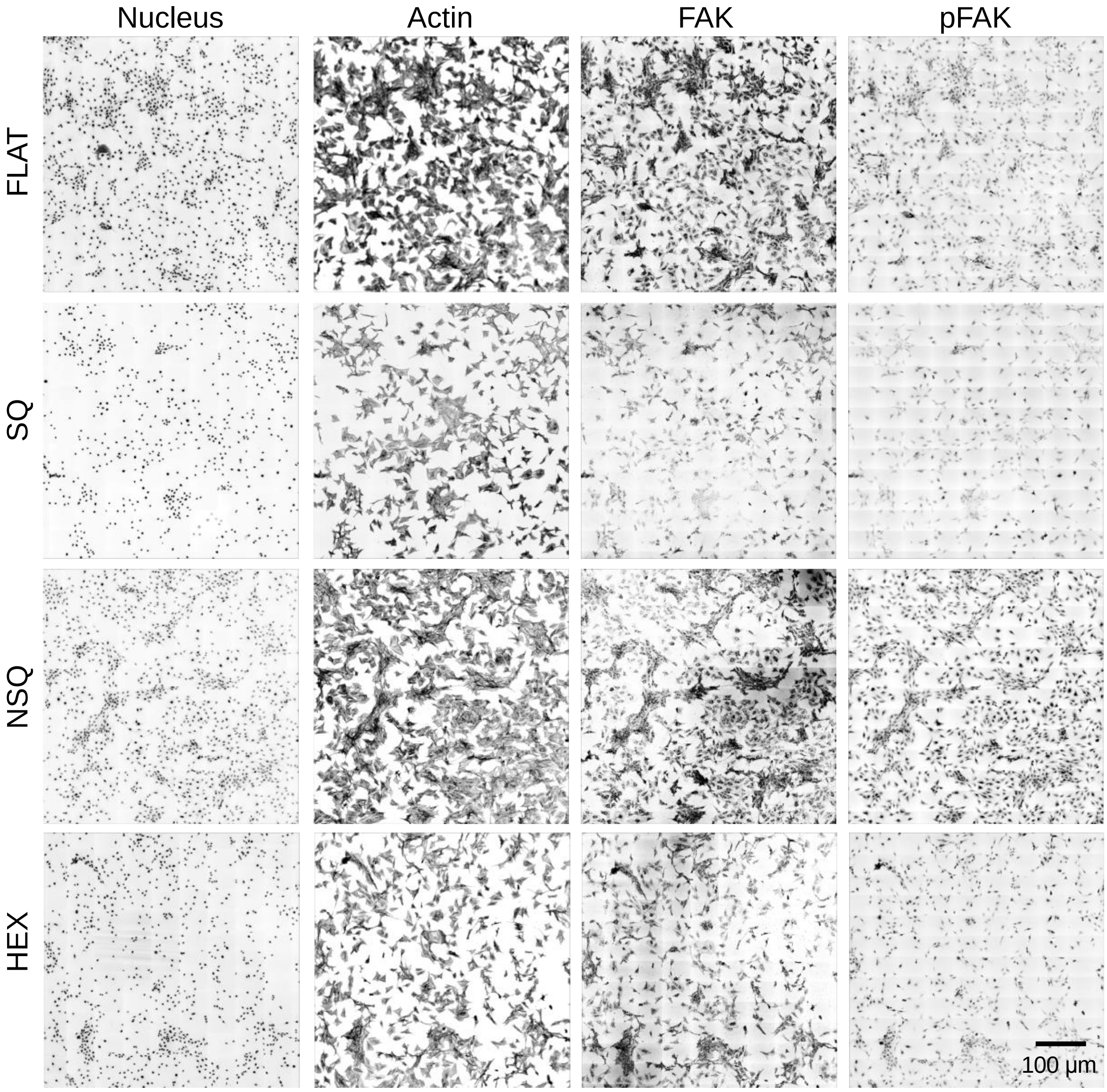

Supplementary Figure 11. Overview of co-culture of pre-osteoblasts and fibroblasts on entire nanotopographies. Images of cells were captured at day 2 using 40X magnification and stitched together to form a tiled image of the entire nanotopography, which was then used for morphome extraction and subsequent analysis. A 2.12 mm x 2.12 mm grid was imaged for each nanotopography. Overview shows homogeneous cell attachment across all nanotopographies. Scale bar = 100 µm.

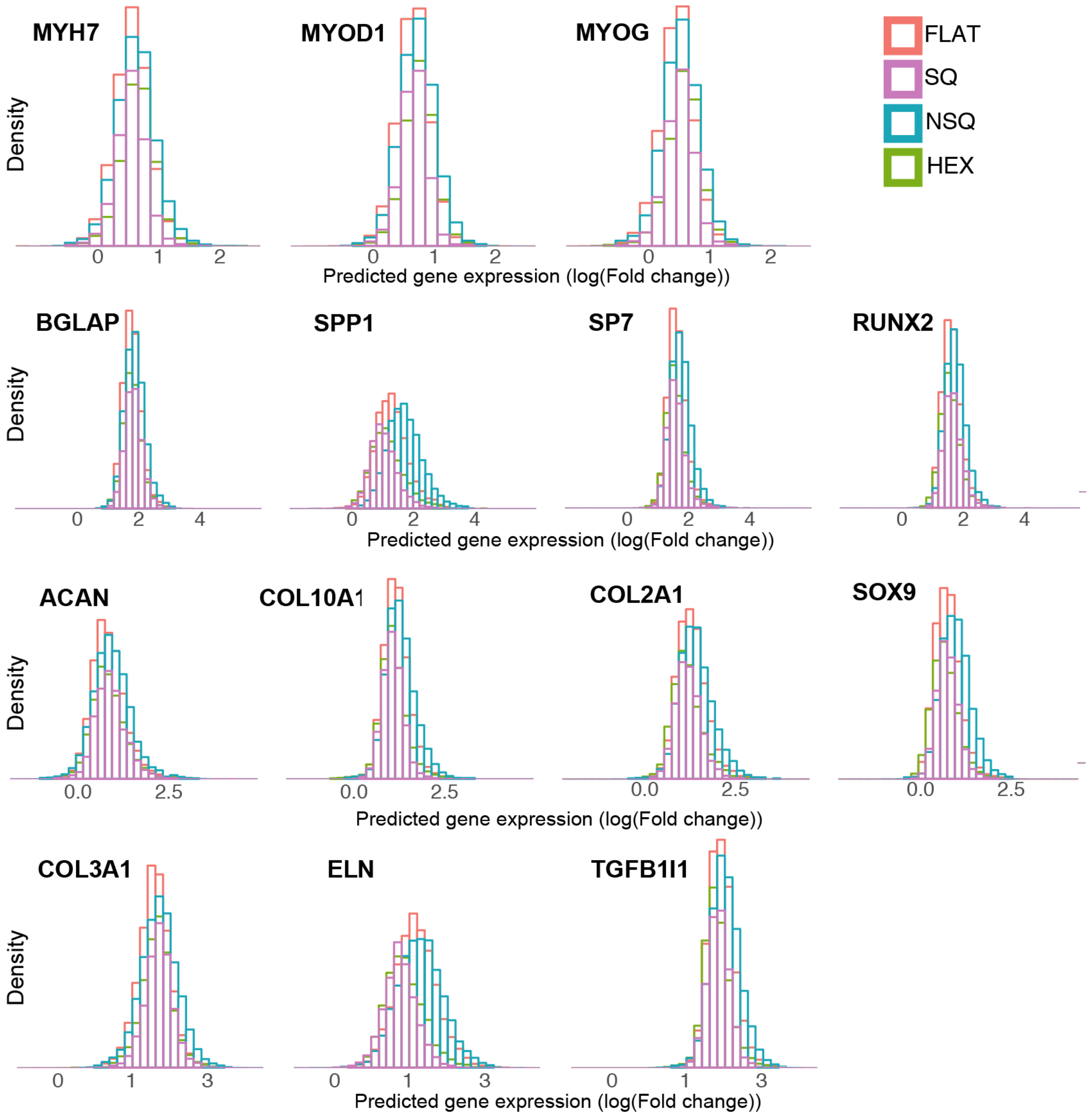

Supplementary Figure 12. Histograms of predicted gene expression from pre-osteoblasts and fibroblasts co-cultured on nanotopographies. The morphome was extracted from all cells on the entire nanotopography. The morphome from the co-culture dataset was then centered to the mean and normalized to the standard deviation of the dataset used to create the predictive linear models. To predict the effect of nanotopographies on cell phenotype, the co-culture morphome was then used as input into the Bayesian linear regression model to obtain qPCR values for 14 different genes. Nanotopographies are color coded, with FLAT denoted in pink, SQ denoted in purple, NSQ denoted in blue and HEX denoted in green.

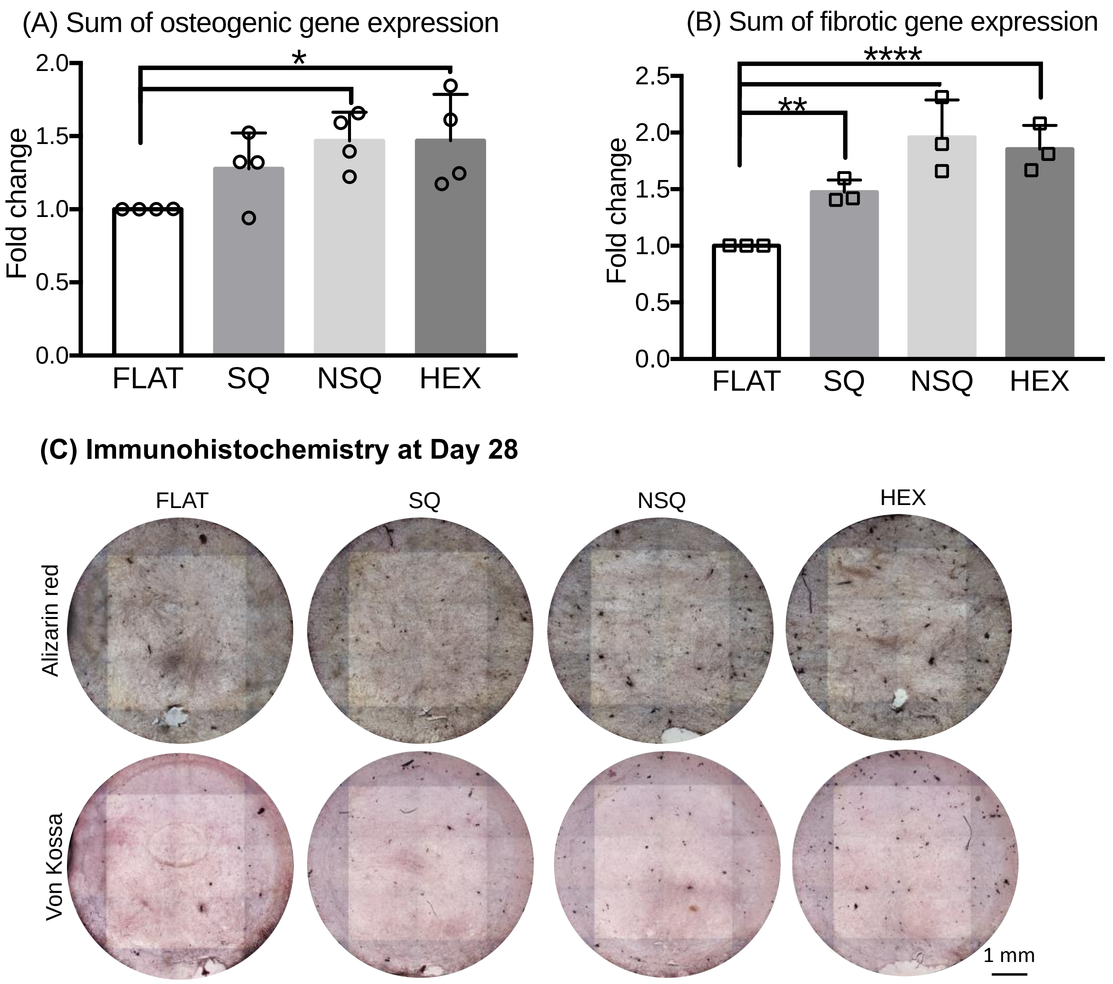

Supplementary Figure 13. Functional response of co-cultured pre-osteoblasts and fibroblasts to nanotopographies. Changes in (A) osteogenic and (B) fibrotic gene expression induced by nanotopography after 7 days of culture. Gene expression was obtained from 2 independent experiments measured twice (n=4). Data shown are mean ± standard deviation, with symbols denoting mean value of each osteogenic (circle) or fibrotic (square) gene. Statistical significance in fold change between FLAT and indicated nanotopography FLAT was calculated using one-way ANOVA with Dunnet's post-hoc test. Significance levels were denoted by * ($p<0.05$), ** ($p<0.01$), *** ($p<0.001$), and **** ($p<0.0001$). (C) Mineralization of cocultured pre-osteoblasts and fibroblasts after 28 days of culture.

Supplementary Table 7. Sequence of primers used for quantitative measurement of gene expression.

Gene	Primer sequence		Amplicon size
18s ribosomal RNA (reference gene)	Fwd	AAGTCCCTGCCCTTTGTACACA	100
	Rev	GATCCGAGGGCCTCACTAAAC	
RUNX2 (Runx2)	Fwd	AAGTGCGGTGCAAACTTTCT	90
	Rev	TCTCGGTGGCTGCTAGTGA	
BGLAP (Osteocalcin)	Fwd	CTGACCTCACAGATGCCAAG	98
	Rev	GTAGCGCCGGAGTCTGTTC	
SPP1 (Osteopontin)	Fwd	TCAGGACAACAACGGAAAGGG	139
	Rev	GGAACTTGCTTGACTATCGATCAC	
SP7 (Osterix)	Fwd	GGTCCAGGCAACACACCTAC	184
	Rev	GGTAGGGAGCTGGGTTAAGG	
SOX9 (Runx2)	Fwd	GGAAGGGAGAGAGAGAGAGAAA	138
	Rev	CGGGATTTAAGGCTCAAGGT	
COL2A1 (Collagen type 2 alpha 1 chain)	Fwd	ACGAAGCGGCTGGCAACCTCA	73
	Rev	CCCTCGGCCCTCATCTCTACATCA	
COL10A1 (Collagen type X alpha 1 chain)	Fwd	TTCTCCTACCACGTGCATGTG	191
	Rev	AGGCCGTTTGATTCTGCATT	
ACAN (Aggrecan)	Fwd	GTGAGGACCTGGTAGTGCGAGTGA	103
	Rev	GAGCCTGGGCGATAGTGGAATATA	
MYOG (Myogenin)	Fwd	GAGACATCCCCCTATTTCTACCA	106
	Rev	GCTCAGTCCGCTCATAGCC	
MYOD1 (Myogenic differentiation 1)	Fwd	CCACTCCGGGACATAGACTTG	109
	Rev	AAAAGCGCAGGTCTGGTGAG	
MYH7 (Myosin heavy chain 7)	Fwd	CTCAAGCTGCTCAGCAATCTATTT	153
	Rev	GGAGCGCAAGTTTGTCATAAGT	
COL3A1 (Collagen type III alpha chain 1)	Fwd	CTGTAACATGGAAACTGGGGAAA	144
	Rev	CCATAGCTGAACTGAAAACCACC	
TGFB1I1 (Transforming growth factor beta 1 induced transcript 1)	Fwd	ATGTCACGGTTAGGGGCTC	83
	Rev	GGCTTGCATACTGTGCTGTATAG	
ELN (Elastin)	Fwd	TGTCCCACTGGGTTATCCCAT	92
	Rev	CAGCTACTCCATAGGGCAATTTC	

(A)

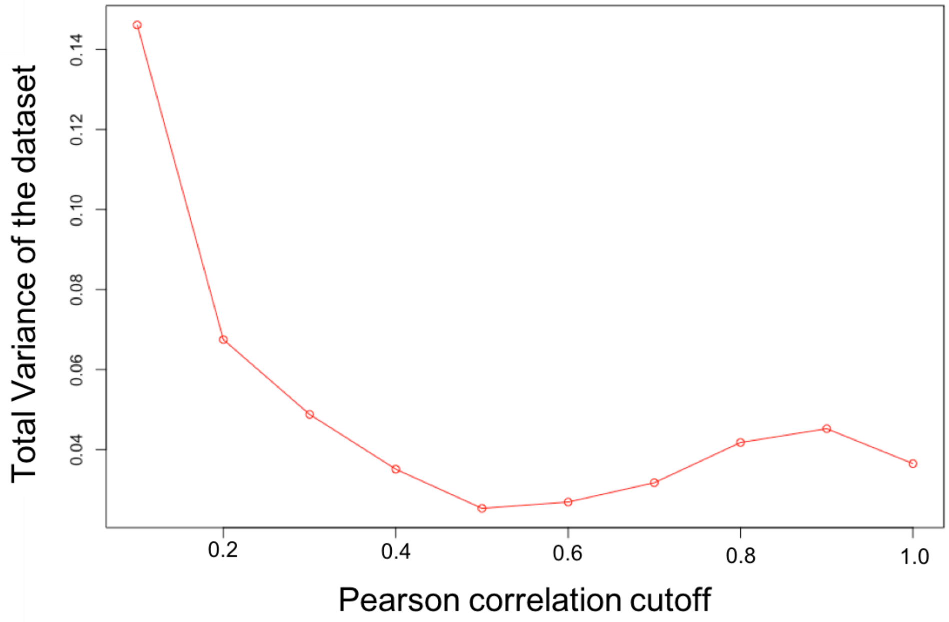

(B)

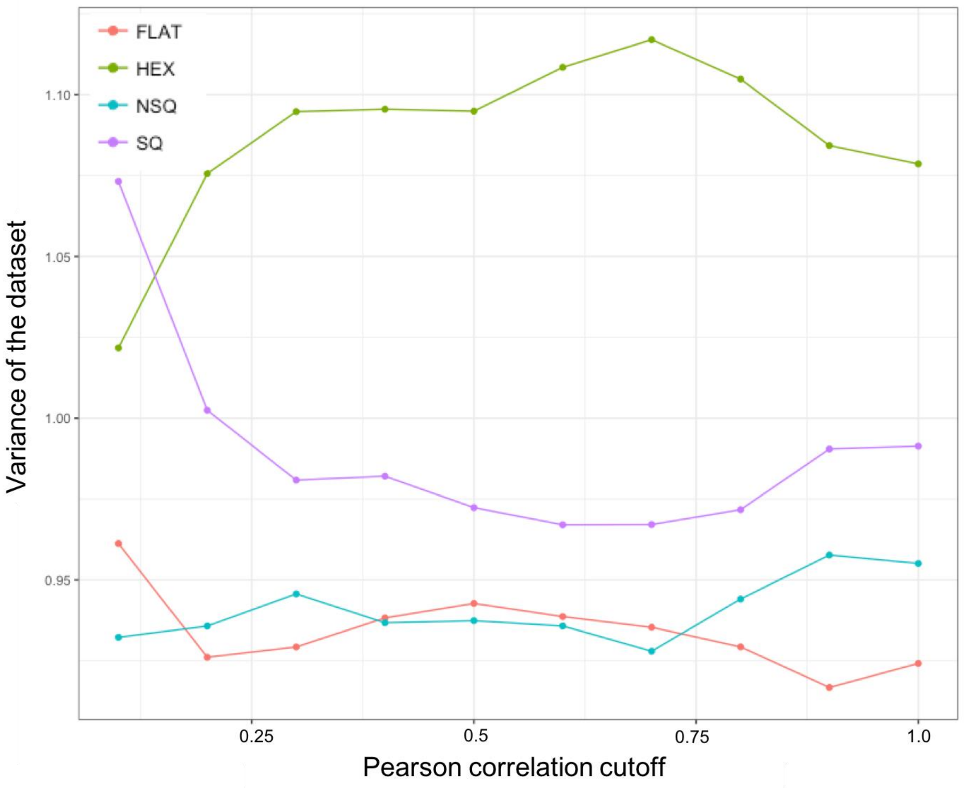

Supplementary Figure 14. Effect of using different Pearson correlation cutoffs to filter the dataset. Total variance from the (A) entire data set or (B) the variance of the topography-specific dataset was measured at different Pearson correlation cutoffs. At a correlation cutoff of 0.9, the data set considerably reduced in feature number without compromising on the dataset variance.

Supplementary Methods

Conventional cell measurements

Images of cells stained against the chromatin, actin, focal adhesion kinase (FAK) and FAK phosphorylated at the Tyrosine 397 site (pFAK) were used to measure cell area, nucleus area, total pFAK/FAK integrated intensity ratio, actin integrated intensity and individual focal adhesion area. The nuclei were segmented using the image of chromatin, the cell body was segmented using the image of actin, while individual focal adhesions were segmented using the image of pFAK. Measurements were taken from distinct cells across 1 independent experiment.

Staining for mechanosensors YAP and TAZ

After 2 days, cells on nanotopographies were fixed and permeabilised as described in the main text (see Materials and Methods). Cells were stained against YAP (Cell Signalling Technologies 4912, 1:70) and TAZ (BD Pharmingen 560235, 1:200) overnight at 4°C then incubated with Alexa Fluor-conjugated secondary antibodies (ThermoScientific, 1:500). Cells were also stained with DAPI and fluorescently conjugated phalloidin (ThermoScientific) to outline the nucleus and the cell body. All substrates were mounted on 0.17 µm thick glass coverslips with ProLong mounting medium (ThermoScientific) and dried overnight at 4°C before imaging.

Biochemical differentiation of musculoskeletal cells

C2C12, MC3T3, chondrocytes and NIH3T3 cells were stimulated biochemically to provide gene expression landmarks of lineage commitment. C2C12 and MC3T3 were biochemically stimulated with established inducers of mature myogenic and osteogenic phenotypes, respectively. Mouse C2C12 myoblasts (ATCC) were cultured in DMEM with 20% FBS and 1% penicillin-streptomycin, and committed into mature myoblastic cells using DMEM supplemented with 2% horse serum and 1% penicillin streptomycin[5,6]. Mouse chondrocytes were cultured in minimum essential medium alpha (MEMα) with nucleosides, ascorbic acid, glutamate, sodium pyruvate supplemented with 10% FBS and 1% penicillin-streptomycin. Mouse MC3T3 cells (ATCC) were cultured in MEMα with nucleosides and L-glutamine without ascorbic acid and supplemented with 10% FBS and 1% penicillin-streptomycin. To commit MC3T3 into mature osteoblasts, MC3T3 media was supplemented with 10 nM dexamethasone, 50 µg per ml ascorbic acid and 10 mM β-glycerophosphate[7,8]. Additionally, we stimulated the commitment of primary chondrocytes and fibroblasts into the chondrogenic and fibrotic lineages through similarly established methods. Chondrocytes

were stimulated by supplementing the growth media with 1% insulin-transferrin-selenium (Gibco), 20 µg per ml ascorbic acid (Sigma Aldrich) and 50 ng per ml bone morphogenetic protein-2 (animal free, PeproTech, Inc)[9]. NIH3T3 were stimulated into the fibrotic phenotype by supplementing the growth media with 5 ng per ml mouse transforming growth factor beta 1 (CellSignaling Technologies)[10-12]. Cells on conventional tissue culture plastic were biochemically stimulated for 16 days, with media change every 2 days. RNA was collected at multiple timepoints and used to measure gene expression, as described in the main text (see Methods section). Gene expression was measured twice from each independent experiment.

Logistic regression

Logistic regression was used as a supervised classification algorithm to separate the morphome according to cell type. A Bayesian logistic regression algorithm was utilised. We consider probability of classification into each cell type *y* to be modelled from the multinomial family[13]. All model parameters were assumed a priori to come from a normal distribution parameterized by mean = 0 and standard deviation = 5. A logistic regression model was trained by excluding from the dataset an entire cell type from one independent experiment (e.g. Chondrocyte from first independent experiment). The held-out dataset was then used as test data to determine classification accuracy. Model convergence was confirmed with the potential scale reduction statistic split-$R_{hat} \geq 1$, effective sample size smaller than total sample size and low autocorrelation. To determine the suitability of the prior distribution, the data regenerated from the prior predictive distribution (i.e. using only model parameters and without seeing any data) closely matched the dataset. For each cell type, classification accuracy was calculated twice (corresponding to two independent experiments). This leave-one-out scheme is critical to measure the true predictive capability of the morphome for classifying cell type without confounding factors from experimental variance[14]. Logistic regression and confusion matrices were created using the *brms*[10] and *caret*[15] package, respectively, on R.

Mineralization study

After 28 days, calcium phosphate deposition was studied using Alizarin red and Von Kossa histological stains. Cells were fixed with 4% paraformaldehyde and stained against 4% Alizarin red (pH 4.2, Sigma Aldrich), or von Kossa (Merck Millipore) and NuclearFast Red (Sigma Aldrich). Samples were imaged at 10X magnification using a color CCD camera.

Supplementary References

1. Kamentsky, L. et al. Improved structure, function and compatibility for CellProfiler: modular high-throughput image analysis software. *Bioinformatics* **27,** 1179–1180 (2011). doi: 10.1093/bioinformatics/btr095
2. Kumar, R. M. & Sreekumar, K. A. survey on image feature descriptors. *Int. J. Comput. Sci. Inf. Technol.* **5**, 7668-7673 (2014).
3. Vega S.L. *et al*. Organizational metrics of interchromatin speckle factor domains: integrative classifier for stem cell adhesion & lineage signalling. *Integr. Biol.* **7**, 435–446 (2015). doi:10.1039/c4ib00281d.
4. Huang K. and Murphy R.F. From quantitative microscopy to automated image understanding. *J. Biomed. Opt.* **9**, 893–912 (2004). doi:10.1117/1.1779233.
5. Quach, N. L., Biressi, S., Reichardt, L. F., Keller, C. & Rando, T. A. Focal Adhesion Kinase Signaling Regulates the Expression of Caveolin 3 and β1 Integrin, Genes Essential for Normal Myoblast Fusion. *Mol. Biol. Cell* **20,** 3422–3435 (2009). doi: 10.1091/mbc.E09-02-0175
6. Clemente, C. F. M. Z., Corat, M. A. F., Saad, S. T. O. & Franchini, K. G. Differentiation of C2C12 myoblasts is critically regulated by FAK signaling. *Am. J. Physiol. Regul. Integr. Comp. Physiol.* **289,** R862–R870 (2005).
7. Quarles, L. D., Yohay, D. A., Lever, L. W., Caton, R. & Wenstrup, R. J. Distinct proliferative and differentiated stages of murine MC3T3-E1 cells in culture: an in vitro model of osteoblast development. *J. Bone Miner. Res.* **7,** 683–692 (1992).
8. Yan, X.Z. *et al.* Effects of continuous passaging on mineralization of MC3T3-E1 cells with improved osteogenic culture protocol. *Tissue Eng Part C Methods* **20,** 198–204 (2014).
9. Perrier-Groult, E., Pasdeloup, M., Malbouyres, M., Galéra, P. & Mallein-Gerin, F. Control of collagen production in mouse chondrocytes by using a combination of bone morphogenetic protein-2 and small interfering RNA targeting Col1a1 for hydrogel-based tissue-engineered cartilage. *Tissue Eng. Part C Methods* **19,** 652–664 (2013).
10. Chen, Y.-L. *et al.* Sorafenib ameliorates bleomycin-induced pulmonary fibrosis: potential roles in the inhibition of epithelial-mesenchymal transition and fibroblast activation. *Cell Death Dis.* **4,** e665 (2013). doi: 10.1038/cddis.2013.154.
11. Ji, Y.D. *et al.* BML-111 suppresses TGF-β1-induced lung fibroblast activation in vitro and decreases experimental pulmonary fibrosis in vivo. *Int. J. Mol. Med.* **42,** 3083–3092 (2018).
12. Negmadjanov, U. *et al.* TGF-β1-mediated differentiation of fibroblasts is associated with increased mitochondrial content and cellular respiration. *PLoS ONE* **10,** e0123046 (2015). https://doi.org/10.1371/journal.pone.0123046
13. Bürkner, P.C. brms: An R Package for Bayesian Multilevel Models Using Stan. *J. Stat. Softw.* **80,** 1–28 (2017). doi: 10.18637/jss.v080.i01
14. Shamir, L. Assessing the efficacy of low-level image content descriptors for computer-based fluorescence microscopy image analysis. *J. Microsc.* **243,** 284–292 (2011).
15. Kuhn, M. Building Predictive Models in R Using the caret Package. *J. Stat. Softw.* **28,** 1–26 (2008). doi: 10.18637/jss.v028.i05
