# Supplementary material for: Predicting gene expression using morphological cell responses to nanotopography
Source: Nat Commun. 2020 Mar 13;11:1384. doi: 10.1038/s41467-020-15114-1 (PMC7070086; doi:10.1038/s41467-020-15114-1)
Supplement: Supplementary file 3 — Description of Additional Supplementary Files [file 41467_2020_15114_MOESM3_ESM.pdf]

Description of Additional Supplementary Files

File Name: Supplementary Data 1
Description: The morphome. The raw morphome data and the list of 624 morphome features measured from all 24 combinations of cell type and nanotopography.

File Name: Supplementary Data 2
Description: Gene expression data. The raw expression levels of 14 genes measured from all 24 combinations of cell type and nanotopography.

File Name: Supplementary Data 3
Description: Morphome features varied across cell type. The raw morphome data consisting of 185 features significantly varied across cell type. One-way ANOVA was used to determine the morphome features that significantly varied across 6 different cell types.

File Name: Supplementary Data 4
Description: Hierarchical clustering of morphome features. Membership of morphome features into specific clusters as determined through silhouette analysis.

File Name: Supplementary Data 5
Description: Fitted parameters for models. Parameters beta were obtained from each Bayesian linear regression model fitted to predict expression level of a single gene using morphome features.
