# Supplementary material for: Predicting gene expression using morphological cell responses to nanotopography
Source: Nat Commun. 2020 Mar 13;11:1384. doi: 10.1038/s41467-020-15114-1 (PMC7070086; doi:10.1038/s41467-020-15114-1)
Supplement: Supplementary file 9 — Reporting Summary [file 41467_2020_15114_MOESM9_ESM.pdf]

nature research

Corresponding author(s): Nikolaj Gadegaard

Last updated by author(s): Jan 15, 2020

Reporting Summary

Nature Research wishes to improve the reproducibility of the work that we publish. This form provides structure for consistency and transparency in reporting. For further information on Nature Research policies, see Authors & Referees and the Editorial Policy Checklist.

Statistics

For all statistical analyses, confirm that the following items are present in the figure legend, table legend, main text, or Methods section.

n/a	Confirmed	
☐	☒	The exact sample size (*n*) for each experimental group/condition, given as a discrete number and unit of measurement
☐	☒	A statement on whether measurements were taken from distinct samples or whether the same sample was measured repeatedly
☐	☒	The statistical test(s) used AND whether they are one- or two-sided<br>*Only common tests should be described solely by name; describe more complex techniques in the Methods section.*
☐	☒	A description of all covariates tested
☐	☒	A description of any assumptions or corrections, such as tests of normality and adjustment for multiple comparisons
☐	☒	A full description of the statistical parameters including central tendency (e.g. means) or other basic estimates (e.g. regression coefficient) AND variation (e.g. standard deviation) or associated estimates of uncertainty (e.g. confidence intervals)
☒	☐	For null hypothesis testing, the test statistic (e.g. *F*, *t*, *r*) with confidence intervals, effect sizes, degrees of freedom and *P* value noted<br>*Give P values as exact values whenever suitable.*
☐	☒	For Bayesian analysis, information on the choice of priors and Markov chain Monte Carlo settings
☒	☐	For hierarchical and complex designs, identification of the appropriate level for tests and full reporting of outcomes
☒	☐	Estimates of effect sizes (e.g. Cohen's *d*, Pearson's *r*), indicating how they were calculated

Our web collection on statistics for biologists contains articles on many of the points above.

Software and code

Policy information about availability of computer code

Data collection	Nucleus and cell segmentation from fluorescent images and measurements of cell characteristics were obtained using CellProfiler v2.4.0.
Data analysis	Multivariate statistical analysis of image features was carried out in R (v3.4.3) using the graphical interface RStudio (v1.0). Aside from the base packages in R, the following packages were used: KMDA (v1.0), gplots (v3.0.1), cluster(v2.0.7-1), corrplot (v0.84), caret (v6.0-81), brms (v2.5.0), ggplot2 (v3.1.0), akima (v0.6-2). Analysis of quantitative PCR values was performed on GraphPad Prism (v7.0a).

For manuscripts utilizing custom algorithms or software that are central to the research but not yet described in published literature, software must be made available to editors/reviewers. We strongly encourage code deposition in a community repository (e.g. GitHub). See the Nature Research guidelines for submitting code & software for further information.

Data

Policy information about availability of data

All manuscripts must include a data availability statement. This statement should provide the following information, where applicable:

- Accession codes, unique identifiers, or web links for publicly available datasets
- A list of figures that have associated raw data
- A description of any restrictions on data availability

Raw data (e.g. gene expression data, morphome data), R workspace data that contains all Bayesian linear regression models, and associated code that support the findings of this study are available in Zenodo with the identifier 10.5281/zenodo.3608197

Field-specific reporting

Please select the one below that is the best fit for your research. If you are not sure, read the appropriate sections before making your selection.

☒ Life sciences ☐ Behavioural & social sciences ☐ Ecological, evolutionary & environmental sciences

For a reference copy of the document with all sections, see nature.com/documents/nr-reporting-summary-flat.pdf

Life sciences study design

All studies must disclose on these points even when the disclosure is negative.

Sample size	No sample size calculation was performed. Two independent experiments were conducted, with each experiment containing at least distinct 75 cells measured without repetition (morphome dataset) or at least 2 repeated measurements from the same sample as technical replicates (gene expression), which contributed heterogeneity in the dataset
Data exclusions	No data were excluded from the analyses.
Replication	Technical and biological replication of qPCR data and image-based data was performed. All technical replicates ensured precision in in the qPCR measurements. Biological replication of both qPCR and image analysis data across 2 independent experiments showed some agreement between replicates thus demonstrating the heterogeneity in biological response captured in single cell and population measurements. However, the heterogeneity in both datasets were taken into account in Bayesian linear regression, where single cell measurements were regressed against gene expression values for each independent biological experiment. That is, measures of central tendency (e.g. mean) across independent biological experiments were not used in machine learning.
Randomization	Separation of the dataset for training and testing of machine learning algorithm was carried out in a completely randomized manner.
Blinding	Blinding of data was not carried out. Supervised machine learning, as carried out in this study, requires that the machine be trained against known or labelled data set to create a predictive regression model.

Reporting for specific materials, systems and methods

We require information from authors about some types of materials, experimental systems and methods used in many studies. Here, indicate whether each material, system or method listed is relevant to your study. If you are not sure if a list item applies to your research, read the appropriate section before selecting a response.

Materials & experimental systems

n/a	Involved in the study	
☐	☒	Antibodies
☐	☒	Eukaryotic cell lines
☒	☐	Palaeontology
☒	☐	Animals and other organisms
☒	☐	Human research participants
☒	☐	Clinical data

Methods

n/a	Involved in the study	
☒	☐	ChIP-seq
☒	☐	Flow cytometry
☒	☐	MRI-based neuroimaging

Antibodies

Antibodies used	pFAK Y397 (Abcam 39967, 1:400 dilution), FAK (ThermoScientific 396500, 1:400 dilution), YAP (Cell Signalling Technologies 4912, 1:70 dilution), TAZ (BD Pharmingen 560235, 1:200 dilution)
Validation	pFAK antibody was validated by testing positive for the immunizing peptide and negative for a non-phosphorylated equivalent peptide. It has also been validated for use in immunofluorescence, as per manufacturer website. The pFAK antibody has been cited in 19 studies, including: https://www.ncbi.nlm.nih.gov/pubmed/29901439 and https://www.ncbi.nlm.nih.gov/pubmed/29763414<br>FAK antibody was verified by immunoprecipitation mass spectrometry with FAK, and has been validated for use in immunofluorescence, as per manufacturer website. The FAK antibody has been used in 1 study: https://www.ncbi.nlm.nih.gov/pubmed/22546345<br>YAP antibody has been validated for use in immunoprecipitation using YAP wildtype and YAP knockout samples. The antibody has also been used in 272 citations: https://www.cellsignal.co.uk/products/primary-antibodies/yap-antibody/4912<br>TAZ antibody has been verified for immunofluorescence, as per manufacturer website. The TAZ antibody has been used in 3 studies: https://www.bdbiosciences.com/eu/applications/research/stem-cell-research/mesenchymal-stem-cell-markers-bone-marrow/human/positive-markers/purified-mouse-anti-taz-m2-616/p/560235<br>Representative images of cells stained using pFAK and FAK antibodies (see Supplementary Figure 1) or YAP and TAZ antibodies (see Supplementary Figure 4) are included as supplementary figures.

Eukaryotic cell lines

Policy information about cell lines

Cell line source(s)	The American Type Culture Collection (atcc.org)
Authentication	None of the cell lines were authenticated
Mycoplasma contamination	None of the cell lines were tested for mycoplasma contamination
Commonly misidentified lines (See ICLAC register)	None of the cell lines are found in the ICLAC register
